# The Dimensionality of the 19-Item Copenhagen Burnout Inventory Adapted for Students: A Bifactor Penalised SEM Analysis

**DOI:** 10.3390/bs16101723

**Published:** 2026-09-22

**Authors:** Pieter Schaap, Melissa Reynell van der Ross

**Affiliations:** Department of Human Resources Management, University of Pretoria, Pretoria 0002, South Africa; reynellvanderross@gmail.com

**Keywords:** Copenhagen Burnout Inventory (CBI), study burnout, personal burnout, peer burnout, hierarchical structure, bifactor model, penalised structural equation modelling (PSEM), alignment loss-function (ALF) priors, study demands, study resources, Study Demands and Resources Scale (SDRS)

## Abstract

Research on the validity of the multidimensional Copenhagen Burnout Inventory (CBI) has predominantly relied on confirmatory factor analysis (CFA), despite concerns regarding inflated factor correlations, local misspecification, and limited subscale distinctiveness. This study evaluated the dimensionality of the 19-item CBI using an adapted student version and cross-sectional data from 1938 South African undergraduates. Three-factor, two-factor, and second-order CFA models were compared with a bifactor penalised structural equation model (PSEM) employing alignment loss function (ALF) priors, auxiliary indices, and criterion validity regression analyses involving study demands and resources. While the three-factor and second-order CFA models demonstrated acceptable global fit, the PSEM local misfit analysis revealed inflated loadings and factor correlations, cross-loadings, and item dependencies that were not sufficiently captured by the global fit indices. The retained bifactor PSEM model demonstrated an excellent fit and indicated a criterion-valid, congeneric general burnout factor. Personal and peer burnout were identified as interpretable residual factors; only peer burnout demonstrated criterion validity, and study burnout was largely accounted for by the general factor. Examples of how to interpret bifactor general-plus-specific burnout scores on the CBI for intervention purposes are provided.

## 1. Introduction

The term burnout was introduced into the psychosocial literature in the mid-1970s by Freudenberger and Maslach (Freudenberger, 1974; Maslach, 1976; Kristensen et al., 2005). It was initially associated with psychological strain, impaired functioning, and reduced well-being among employees engaged in “people work” but has since been extended to the broader occupational context and student burnout. Burnout is generally characterised by physical, emotional, and mental exhaustion, often linked to prolonged emotionally demanding work (Schaufeli & Greenglass, 2001). Although burnout research and intervention remain dominated by the Maslach Burnout Inventory (MBI), debates persist regarding its structure and psychometric properties. The core limitation of the MBI is the confounding of exhaustion with attitudinal components, including cynicism, depersonalisation as a coping strategy, and reduced accomplishment as a consequence of burnout (Kristensen et al., 2005).

The Copenhagen Burnout Inventory (CBI) aligns more closely with traditional and contemporary definitions of burnout by emphasising physical and emotional exhaustion across personal, work-related, and client-related domains (Kristensen et al., 2005). Since its development, the 19-item CBI, which comprises personal, work-related, and client-related subscales, has been widely accepted. Its psychometric validity has been confirmed across various occupations, including nursing (Montgomery et al., 2021; Tran et al., 2023), teaching (Milfont et al., 2008), social work (Walters et al., 2018), home care provision (Jeon et al., 2019), and pharmacies (Youssef et al., 2022). The attributional nature of CBI items allows for adaptation across various occupations (e.g., patient burnout) and educational and student populations (Kristensen et al., 2005; Milfont et al., 2008). Like workers, students experience sustained workloads, evaluative pressure, limited resources, and interpersonal demand. The 19-item CBI can be adapted for students by reframing work burnout as coursework burnout and client burnout as colleague or peer burnout, and it can be extended to include teacher burnout (Campos et al., 2013; Stein & Sibanda, 2016). These adaptations preserve most of the CBI’s core structure, while enhancing its ecological relevance.

Despite the widespread use of the 19-item CBI and its strong psychometric foundation, studies have primarily focused on exploratory and confirmatory factor analyses (EFAs and CFAs) across various samples. The literature also includes criterion-related studies, translations, and invariance tests for gender and language. EFA and CFA commonly identify three-factor, second-order, and two-factor models. Although CFA models usually fit well, they often lack local misspecification checks. Many studies have also followed the questionable practice of sequentially freeing correlated data-driven residuals to improve model fit. Evidence consistently shows high factor correlations, especially between personal and work- or study-related burnout (r > 0.80), raising questions about discriminant validity and the three-factor structure (Moser et al., 2023; Rocha et al., 2020; Živanović et al., 2021). In contrast, burnout related to clients in occupational settings and burnout associated with peers or teachers in academic settings exhibited moderate correlations with personal and work- or study-related burnout (r = 0.50–0.60 in occupational settings; r = 0.35–0.55 in academic settings) (Campos et al., 2013; Walters et al., 2018). This indicates a stronger discriminant validity.

Although CFA studies on the CBI are commendable, reliance on conventional CFA findings may be questioned, given their limitations and the need for more refined psychometric analyses (Pretorius & Padmanabhanunni, 2025). A key limitation is that CFA models can give the illusion of good model fit when unrealistic zero cross-loadings and zero residual correlations are imposed on imprecise self-report burnout measures, leading to substantive local misspecifications that go undetected. These misspecifications may be absorbed into inflated factor loadings or factor correlations, thereby masking local misfit despite acceptable global fit—a phenomenon described as the model fit paradox (Alamer, 2022; Savalei & Huang, 2025; Wang et al., 2025). These limitations could affect the validity of scale scores or conclusions drawn from relationships between burnout and external variables, such as study demands and resources or personal well-being and work engagement. Moreover, correlated multidimensional factor models are rarely sufficient to establish subscale reliability when there is evidence of shared variance among factors (Rodriguez et al., 2016a). This shared variance may cloud the interpretation of factors as distinct variables and has not been adequately addressed for the CBI.

Accordingly, the central research question is not only whether the adapted CBI is multidimensional, but also whether its specific factors remain sufficiently reliable, distinct, interpretable, and criterion-valid after accounting for the shared variance attributable to a putative general burnout factor. Bifactor analysis is an effective tool for partitioning common and specific sources of variance in the covariance matrix and, when theoretically justified, for evaluating whether a general factor, specific factors, or both support a defensible measurement interpretation (Rodriguez et al., 2016a). The recent application of bifactor exploratory structural equation modelling (ESEM) to the Burnout Assessment Tool (BAT) provides a relevant precedent for using theory-supported bifactor models to examine the strength of a general burnout factor, the added value of specific factors, and the scoring implications of the resulting measurement structure (De Beer et al., 2026).

Theoretically, there is substantial support for a general burnout factor and domain-specific facets that form a hierarchical structure. This structure represents burnout as a coherent syndrome with distinguishable symptom domains (Schaufeli et al., 2020), as empirically demonstrated in bifactor models of the MBI and BAT (Aguayo-Estremera et al., 2024; De Beer et al., 2026). To our knowledge, a hierarchical structure comprising a general factor alongside specific factors has not yet been tested for the 19-item CBI, highlighting an important gap in the literature that this study addresses. Therefore, this study examined whether the CBI exhibits a hierarchical bifactor structure comprising three statistically interpretable residual factors in addition to a broad general factor.

To address the question of the CBI structure, we used penalised bifactor SEM (PSEM) rather than bifactor ESEM. PSEM improves parsimony by distinguishing minor from significant cross-loadings and using algorithms to identify cross-loadings and correlated residuals associated with misspecification, while maintaining model fidelity (Asparouhov & Muthén, 2024). To our knowledge, PSEM has not yet been applied to burnout measures. PSEM with an alignment loss function (ALF) prior is superior to ESEM target rotation because ALF priors favour parsimony. PSEM and ALF priors concentrate misspecification in a few important cross-loadings that depart from zero, whereas ESEM target rotation tends to spread the error across all targeted loadings when the structure is imperfect. Consequently, PSEM yields less biased and more robust estimates than ESEM (Asparouhov & Muthén, 2024). We assessed the strength and interpretability of factors in the bifactor model using auxiliary indices, including percentage of uncontaminated correlations (PUC), explained common variance (ECV), Omega hierarchical (ωH), value-added ratio (VAR), and sum of squared factor loadings (SS_λ_) (Petras & Meiser, 2024; Rodriguez et al., 2016b). These indices included new criteria not previously used in burnout measures to evaluate the stability, interpretability, and added value of specific or residual factors relative to the general factor (Dueber & Toland, 2023).

However, structural evidence for the distinctiveness and interpretability of factors in the bifactor model should be complemented by evidence of external validity (Ferrando & Lorenzo-Seva, 2019). We used the Study Demands unidimensional scale and the Resources Scales’ bifactor model (van Zyl et al., 2024), incorporating associations not previously included in CBI validation studies. These scales assess workload, growth opportunities, lecturer and peer support, and access to information. These constructs are considered antecedents of students’ academic work or study burnout and peer burnout according to the job demands–resources model (Bakker & Demerouti, 2017). The model reflects the process in which a lack of study resources leads to an inability to cope with and navigate high study demands associated with burnout (Bakker & Mostert, 2024). By analysing these relationships within a bifactor model, we assessed the contributions of the general and residual factor dimensions to the interpretation and use of factor scores. To obtain robust estimates of the relationships among factor variables, we isolated the structural model from the measurement models using a two-phase approach (Rosseel, 2020), with derived plausible values (PVs) serving as latent variables or true scores (Asparouhov & Muthén, 2010).

This study adopts a substantive–methodological synergy approach by applying newly developed statistical methods to address substantive questions and advance burnout theory (Hofmans et al., 2021). Our main objective was to determine whether the CBI exhibits a hierarchical bifactor structure comprising a broad general factor and three distinct, interpretable, and theoretically meaningful residual factors that are criterion-valid.

More specifically, we aimed to achieve our main objective by doing the following:

(a) Testing competing CBI measurement models reported in the literature: three-factor CFA, two-factor CFA, and second-order CFA;
(b) Testing a three-factor PSEM model with cross-loadings using PSEM with ALF priors;
(c) Estimating a bifactor PSEM model with cross-loadings and correlated residuals using ALF priors to identify meaningful local misspecifications;
(d) Incorporating empirically and theoretically supported minor cross-loadings and substantively vetted correlated residuals in the restricted bifactor PSEM CBI model;
(e) Evaluating the strength of the general burnout factor and the distinctiveness, stability, and interpretability of residual-specific factors that provide additional value over the general factor using bifactor auxiliary indices and newly developed criteria;
(f) Clarifying whether scoring should emphasise a total burnout score, specific subscale scores, or a general-plus-specific interpretation, and whether unit-weighed and optimal-weighted scoring are plausible;
(g) Assessing the criterion-related validity of the CBI bifactor PSEM model using the Study Demands and Study Resources Scales (SDRS) to determine the relative contribution of the general factor versus the specific factors in the relationship.

This study offers substantive and methodological contributions. Substantively, it is among the first to examine whether the adapted CBI primarily measures a general burnout factor for students and whether personal, academic/study, and peer-related residual factors add value internally and externally. Methodologically, it goes beyond CFA, second-order CFA, and two-factor models by incorporating bifactor modelling, PSEM with cross-loadings, local misspecification analysis, new criteria for bifactor auxiliary indices, and criterion-validity analysis using the SDRS. The hypothesis that the CBI is a hierarchically structured bifactor model comprising a general, congeneric burnout factor and three stable, interpretable residual-specific factors, and that it has criterion validity, was only partially supported. The congeneric general burnout factor in the bifactor model was supported by the data and showed criterion validity. While both peer burnout and personal burnout met all criteria for the bifactor model to be considered distinct residual-specific factors, only peer burnout demonstrated criterion validity. Further research is required to verify the criterion validity of the personal burnout factor. The data did not support the three-factor, second-order, or two-factor CFA models, challenging earlier findings. These models appear compromised by cross-loadings, correlated residuals, and shared variance, which inflate primary factor loadings and produce high factor correlations. Unidimensional and essentially unidimensional bifactorial models assuming reliable unit-weighted scoring were also unsupported. However, the congeneric bifactor model supports optimal-weighted rather than unit-weighted scoring. Scoring and interpretation options for the bifactor model are outlined.

## 2. Literature Review

The following sections adopt a substantive methodology synergy framework, beginning with substantive (theoretical) considerations and then presenting aligned methodology considerations to generate new insights into the subtle nuances of the CBI’s dimensionality.

### 2.1. Substantive Considerations

In this section, we provide an overview of what burnout entails in occupational and student contexts, related measures of burnout, and details on the CBI constructs. We define and explain student burnout in higher education from a demand-resources perspective, which served as a criterion-relevant, ascendant variable and was operationalised in the student demands and resources measure. Lastly, we provide previous research findings on the dimensionality and validity of the CBI.

### 2.2. Understanding Burnout: Occupational, Student and CBI

Although the definition of burnout varies across contexts and measures, the World Health Organization (2019) describes it as a syndrome rather than a medical condition. It can be defined as ongoing workplace stress that has not been effectively managed. It is characterised by a sense of energy depletion or exhaustion, a growing detachment from one’s job, and feelings of negativity or cynicism towards one’s work, with the construct’s core rooted in a lack of energy (Gaspar et al., 2025). The latter conceptualisation of burnout coincides with the constructs measured in the Maslach Burnout Inventory (MBI), which has been criticised for confounding the core syndrome of exhaustion with distinct attitudinal components, such as cynicism, depersonalisation as a coping strategy, and reduced performance, as a consequence of burnout (Kristensen et al., 2005). Similarly, student burnout is characterised by three primary symptoms: exhaustion due to academic demands, a cynical attitude towards learning and academic goals, and feelings of inadequacy leading to decreased performance, which is synonymous with job-related burnout (Jagodics & Szabó, 2022; Maroco & Campos, 2012). To measure student burnout, work-burnout measures have been adapted for student use, including the MBI–Student Survey (MBI–SS), the Copenhagen Burnout Inventory–Student Survey (CBI–SS), and the Oldenburg Burnout Inventory–Student Survey (OLBI–SS) (Maroco & Campos, 2012).

As with the MBI, Schaufeli et al. (2020, p. 4) operationalised BAT-C as a broad conceptualisation of burnout as an ‘inability’ characterised by exhaustion, impaired emotional and cognitive control, or ‘unwillingness’ characterised by mental distancing. They recognised exhaustion as the most obvious symptom of burnout, although impaired emotional and cognitive regulation due to low energy is also a core element. In addition, mental distancing or withdrawal from the job is a coping strategy for dealing with feelings of exhaustion. In the BAT-S, Schaufeli et al. (2020) operationalised depression, psychological distress, and psychosomatic complaints as secondary dimensions of burnout; however, they recognised these as atypical, as they also appear in other mental disorders. More importantly, Schaufeli et al. (2020) emphasised that burnout is a syndrome comprising related symptoms that constitute a single underlying psychological condition. This is represented in the BAT-C as a general factor and four specific factors within a hierarchical second-order SEM model, and later in a bifactor model (De Beer et al., 2026; Redelinghuys & Morgan, 2023).

Within the framework of the CBI, Kristensen et al. (2005) narrowed burnout to its core, defined as ‘a state of physical, emotional, and mental exhaustion that results from long-term involvement in work situations that are emotionally demanding’. They argued that burnout is not merely general tiredness; rather, its meaning depends on whether exhaustion is experienced generally, attributed to work, or attributed to work with clients. More specifically, treating the attribution of this exhaustion as work-related is central to the construct. The CBI therefore operationalises burnout through three domain-specific exhaustion scales: personal burnout, work-related burnout, and client-related burnout, which, as expected, are differentially correlated with fatigue and psychological well-being across occupational groups (Kristensen et al., 2005). The personal burnout scale captures general physical and psychological exhaustion, regardless of its source. The work-related burnout scale captures exhaustion specifically attributed to work; in student adaptations, this refers to academic work or studying (Maroco & Campos, 2012). The client-related burnout scale captures exhaustion attributed to sustained interpersonal demands from clients or service recipients and can be adapted to relevant academic relational targets, such as peers, lecturers, or students (Maroco & Campos, 2012). In conclusion, the CBI, with its focus on fatigue, is a key instrument for burnout measurement and can be readily adapted across contexts, including academic settings, as reflected in more recent student versions (Bolatov et al., 2021; Krishnappa et al., 2024).

### 2.3. Student Burnout in Higher Education: A Demand-Resources Perspective

In higher education, student burnout can be conceptualised as the academic analogue of occupational burnout because studying constitutes sustained, goal-directed, and evaluative work. Student burnout is typically characterised by exhaustion and reduced academic efficacy due to study demands that chronically exceed the resources available to manage them (Bakker & Mostert, 2024; Jagodics & Szabó, 2022). Study demands include workload, deadlines, examinations, emotionally demanding academic expectations, uncertainty, and sustained cognitive effort. Study resources include lecturer support, peer support, constructive feedback, autonomy, meaningful learning opportunities, and institutional support. Jagodics and Szabó (2022) found that student burnout was positively associated with university demands and negatively associated with resources, supporting the relevance of the demand–resource framework in higher education. South African evidence similarly shows that high study demands and inadequate study resources are associated with burnout, while resources support engagement and well-being (Mokgele & Rothmann, 2014).

### 2.4. Dimensionality of the CBI and Published Validity Studies

The original 19-item Copenhagen Burnout Inventory (CBI) remains the primary basis for comparison in validation studies, conceptualising burnout as exhaustion across three domains: personal (PB), work-related (WB), and client-related (CB). Kristensen et al. (2005) did not conduct EFA or CFA because the scales were retained on theoretical grounds but reported strong internal consistency (α = 0.85–0.87), a substantial PB–WB correlation (r = 0.72), and moderate PB–CB and WB–CB correlations (r = 0.46; r = 0.61) for raw scores. Criterion validity was supported by associations with SF-36 vitality and mental health, sickness absence, sleep problems, and intention to quit.

Subsequent CFA studies support the CBI’s three-factor structure, sometimes a second-order structure, with selective support for two-factor or abbreviated versions across English, translated, and occupational applications. Translated versions exist in multiple languages, including Chinese/Taiwanese, Colombian Spanish, Latvian, Russian, Vietnamese, Italian, Peruvian Spanish, and Arabic (AL-Qadri et al., 2026; Amaya-Arias, 2026; Breña et al., 2022; Cerela-Boltunova et al., 2025; Fiorilli et al., 2015; Fong et al., 2014; Nguyen et al., 2025; Seisembekov et al., 2025; Yeh et al., 2007). Overall, the translated and English versions generally exhibit similar model fit, reliability, factor structures, and factor correlation patterns, although universal invariance is not assumed (Johnson, 2024; Kováč & Lisá, 2025; Montgomery et al., 2021; Thrush et al., 2021). See Supplementary Materials Tables S1 and S2 for a detailed overview and trend summary of the EFA/CFA models tested in the CBI validation literature.

Model fit was acceptable to good (CFI: 0.92–0.96; RMSEA: 0.065–0.09), although four to six correlated residuals were often freed to obtain fit. For example, Walters et al. (2018) freed four: PB1–PB2, WB5–WB6, CB1–CB2, and CB5–CB6. Generally, reliabilities were high (α/ω ≈ 0.87–0.91); PB–WB correlations were strong (r ≈ 0.79–0.97), while PB–CB and WB–CB were moderate (r ≈ 0.48–0.68). Criterion-related validity was supported by medium-to-large associations with distress, work demands, well-being, job satisfaction, stress, and depression (Cordero-Franco et al., 2022; Fong et al., 2014; Lapa et al., 2018; Seisembekov et al., 2025; Yeh et al., 2007). Li et al. (2025)’s abbreviated version of the CBI for emergency medicine showed reliable 2-factor, 10-item scores, although structural validity evidence was mixed. Additionally, the reverse-scored WB item regarding insufficient energy for family and friends showed weak loadings and was recommended for exclusion (Amaya-Arias, 2026; Fong et al., 2014; Rocha et al., 2020).

Student versions adapted work- and client-related burnout to study-, peer/colleague-, and teacher/lecturer-related contexts, producing 25-item CBI-S/CBI-SS versions that were generally retained as four-factor models (Bolatov et al., 2021; Campos et al., 2013; Oluwadiya et al., 2024; Wongtrakul et al., 2021). In addition to the original PB factor, the adapted factors included study burnout (SB), colleague/peer burnout (CB^S^), and teacher burnout (TB). These showed acceptable CFA fit, good reliability, and high PB–SB correlations (r ≈ 0.66–0.97). However, the CB^S^ and TB factors showed better discrimination than the original client-related factor. PB–CB^S^ and SB–CB^S^ correlations were modest (r ≈ 0.30–0.45), whereas PB–TB and SB–TB associations were stronger (r ≈ 0.40–0.55). Associations with depression, anxiety, academic self-efficacy, and study satisfaction support the criterion-related validity of the student versions (Bolatov et al., 2021; Campos et al., 2013; Oluwadiya et al., 2024).

Collectively, these findings provide prima facie support for multidimensional CBI scoring. However, the consistently high PB–WB/SB overlap and acceptable second-order models also suggest that a hierarchical structure comprising a general burnout factor and residual-specific factors is both plausible and psychometrically important (Amaya-Arias, 2026; Lapa et al., 2018; Maroco & Campos, 2012; Milfont et al., 2008; Walters et al., 2018). High PB–WB correlations in CFA models may partly result from inflated factor correlations due to zero cross-loadings. Additionally, the common practice of freeing correlated residuals to enhance model fit may mask the effects of local item dependencies, which may appear as mis-specified cross-loadings. Therefore, bifactor models offer more appropriate frameworks for distinguishing general and specific sources of variance in hierarchical structures and for PSEM to address the effects of minor cross-loadings on factor structures (Asparouhov & Muthén, 2024; Rodriguez et al., 2016b; Swami et al., 2023). This notion of a hierarchical bifactor structure is further supported by theory and empirical work on general and specific burnout factors, as reflected in related measures such as the MBI and BAT-C (Aguayo-Estremera et al., 2024; De Beer et al., 2026; Redelinghuys & Morgan, 2023; Schaufeli et al., 2020).

However, Kristensen et al. (2005) did not conceptualise the CBI as a hierarchical statistical model or assume that its three scales would emerge as statistically distinct latent factors. Their formulation was theoretically grounded, with physical, emotional, and mental exhaustion constituting the common core of burnout, and the three scales differentiated by the situational attribution of the same exhaustion. This conceptualisation is compatible with a bifactor representation in which common exhaustion variance coexists with domain-specific attributional variance. Consistent with this interpretation, the accumulated empirical evidence reviewed above indicates substantial shared variance among the CBI dimensions and supports hierarchical representations, while the strength and configuration of general and specific factors remain empirical questions.

### 2.5. Methodological Considerations

This section introduces modern SEM techniques that extend beyond traditional CFA models. Specifically, these methods help detect and resolve model misspecifications that affect fit and disentangle the sources of variance that shape the model’s structure and interpretation.

### 2.6. PSEM for Identifying and Resolving Independent Cluster CFA Models’ Misspecifications

Independent cluster CFA (IC-CFA) models can show excellent global fit indices (e.g., CFI, TLI, RMSEA), even when the true data structure contains non-zero cross-loadings, because these misspecifications may be absorbed by the model, resulting in inflated factor correlations or residual covariances (Alamer, 2022; Savalei & Huang, 2025; Wang et al., 2025). Even a single substantive local misspecification can distort parameter estimates across the model while remaining largely undetected by global fit indices (Ropovik, 2015). PSEM and ESEM address this limitation by allowing cross-loadings to be estimated, thereby providing a more realistic representation of complex measurement structures (Alamer, 2022; Asparouhov & Muthén, 2024).

The PSEM model with ALF priors can serve as a local parameter misspecification or diagnostic tool, effectively distinguishing cross-loadings and correlated residuals that substantively depart from zero (e.g., >0.30) from those that do not (e.g., <0.10), which IC-CFA global fit indices may be insensitive to. Freeing all parameter estimates in the CFA models results in an unidentified model, whereas in PSEM, all cross-loadings and residual correlations can be estimated simultaneously using ALF priors. In PSEM, ALF priors act as penalty functions that help identify and resolve otherwise unidentified model parameters. It favours parsimonious, sparse, and interpretable solutions by concentrating deviations in relatively few parameters rather than spreading misfit across the model. Moreover, PSEM offers potential model modification options for cross-loadings and correlated residuals by identifying and resolving all model misspecifications in a single step with ALF priors, rather than IC-CFA’s much-criticised, data-driven, sequential process of freeing parameters to improve model fit (Asparouhov & Muthén, 2024).

Misspecified cross-loadings and correlated residuals in IC-CFA, arising from the fallibility of human responses and systematic errors due to the theoretical overlap between factors, may bias theoretical conclusions, reliability estimates, and subsequent structural models. In contrast, PSEM explicitly accommodates cross-loadings and correlated residuals and can distinguish meaningful misspecifications from irrelevant white noise, as reflected in multiple near-zero, statistically nonsignificant parameter estimates. These negligible parameters can subsequently be set to zero in a simplified follow-up or final model, allowing minor cross-loadings and isolated correlated residuals where justified, particularly when the measure contains many items (Asparouhov & Muthén, 2024). For instance, justified correlated residuals may indicate method effects, such as item redundancies, parallel wording, and item-adjacency effects, which are replicable across samples and attributable to fallible human responses (e.g., response sets). However, meaningful cross-loadings and correlated residuals may also indicate model misfit due to missing, extra, redundant, or residual factors, warranting a review of the model (Asparouhov et al., 2015).

Overall, PSEM provides a viable means of optimising model parsimony while maintaining model fidelity, resulting in high-accuracy and low-bias estimates. Overfitting was constrained by monitoring the ALF penalty ratio, which typically ranged between 0.01 and 0.10. Using PSEM with ALF priors preserves the theory-based framework of the measurement model and improves its alignment with empirical data. It also reduces reliance on arbitrary post hoc adjustments and provides a clearer basis for distinguishing between cross-loadings and correlated residuals that are merely minor noise and those that reflect a meaningful local misfit (Asparouhov & Muthén, 2024).

A further advantage of ALF priors over Bayesian SEM (BSEM), LASSO, and ridge priors is that the regularisation process is determined by the structure of the model rather than by data-dependent prior-variance choices. ALF priors do not require complex informed prior settings or extensive sensitivity analyses, which are often necessary for BSEM. Instead, they operationalise the conceptual aim of finding the simplest adequately fitting model, analogous to an interpretable geomin-rotated simple-structure solution in EFA. ALF priors are also more aggressive in rewarding parsimony: they set as many negligible parameters as possible to zero while allowing substantively meaningful deviations from zero to emerge as local mis-specifications. In contrast, BSEM and ridge-type normal priors tend to distribute small mis-specifications across the model. Thus, PSEM with ALF priors provides a parsimonious, diagnostically useful, and comparatively less biased alternative to the ESEM target rotation, BSEM, ridge, and LASSO approaches using normal priors (Asparouhov & Muthén, 2024).

In summary, PSEM with ALF priors offers powerful capabilities for estimating complex and unidentified models but comes with limitations, including increased computational complexity and the need to carefully monitor penalty settings and monitor convergence to address overfitting. It functions partly as an exploratory procedure and can be difficult to interpret because the ALF prior favours parsimony over simply improving the global model fit (Asparouhov & Muthén, 2024).

### 2.7. Bifactor Models for Resolving Common Variance Effects on Multi-Dimensional CFA Models

Correlated multi-factor CFA models may, at face value, produce well-defined subscales and acceptable global fit, yet they obscure the common variance or the global factor underlying these factors (Reise et al., 2010). Moreover, these subscales may partly reflect residual covariance within redundant item clusters due to content overlap, parallel wording, or response sets arising from the close ordering of same-factor items in blocked formats (Loiacono & Wilson, 2020). Under these conditions, the resulting factors may reflect inflated specifics and method effects rather than substantively distinct constructs because most of the meaningful shared variance between items is captured by the general factor, while residual item-cluster covariance is too readily interpreted as meaningful subscales (Rodriguez et al., 2016a).

Bifactor modelling provides a more rigorous test of the assumption of the multifactor CFA model by partitioning each item’s variance into a general factor and one residual-specific factor, thereby allowing the broad construct and the narrower domains to compete directly for common variance. This allows determining whether a measure is essentially unidimensional, multidimensional, or hierarchical, with a strong general factor and interpretable specific factors (Rodriguez et al., 2016a). Unlike second-order CFA, where the general factor influences items indirectly via first-order factors, the bifactor model directly estimates item loadings on both the general and specific factors. This approach avoids the proportionality constraint of second-order models, which requires that items within a domain contribute fixed proportions to both the general and specific variances, a requirement that is rarely met (Brunner et al., 2012; Markon, 2019).

Bifactor modelling extends the interpretation of model fit beyond CFI, TLI, and RMSEA because a good global fit does not indicate whether subscales retain reliable unique variance after the general factor has been removed. Therefore, bifactor auxiliary indices were used (Dueber, 2017; Dueber & Toland, 2023; Petras & Meiser, 2024; Rodriguez et al., 2016a, 2016b): Omega hierarchical (ωH) estimates reliable total-score variance due to the general factor; Omega (ωS) estimates subscale reliability due to general and specific sources combined; Omega hierarchal specific (ωHS) estimates reliable subscale variance uniquely due to the specific factor; explained common variance (ECV) estimates the proportion of common variance explained by the general factor; explained common variance specific factor (ECVss) estimates specific-factor common variance after removing the general factor; percentage of uncontaminated correlations (PUC) indicates the proportion of correlations reflecting only the general factor; factor determinacy (FD) indicates factor-score determinacy; construct replicability (H) estimates reliability of an optimal-weighted composite; absolute relative parameter bias (ARPB) estimates bias when bifactor data are treated as unidimensional; value-added ratio (VAR) evaluates the added value of the specific factor; and sum of squared factor loadings (SS_λ_) estimates specific-factor strength and stability. These indices, especially when interpreted using the recent criteria of Dueber and Toland (2023) and Petras and Meiser (2024), provide stronger evidence for score interpretation than global fit alone.

Auxiliary indices provide evidence of the extent to which a general factor can be considered essentially unidimensional, along with the reliable and stable added value of the residual factors (Rodriguez et al., 2016b). Conventional cut-off criteria for essentially unidimensionality assume approximately homogeneous factor loadings and unit-weighted reliable scores, which may not be applicable across all bifactor models. When factors in a correlated factor model are not equally correlated, the loadings of these items on the general factor of a bifactor model may be heterogeneous, indicating a congeneric factor model, and an optimally weighted scoring method is preferred (McNeish & Wolf, 2020). The CBI, based on previous studies, shows uneven factor correlates and is consistent with this study, which supports the congeneric model (Bolatov et al., 2021; Campos et al., 2013; Oluwadiya et al., 2024; Wongtrakul et al., 2021).

A limitation of bifactor models is their susceptibility to overfitting, in which global fit indices indicate an excellent fit, while the model reflects sample-specific idiosyncrasies, undermining generalisability (Swami et al., 2023). However, this risk can be mitigated through theory-guided modelling using large study samples, triangulating indices of global fit with local misfit and bifactor auxiliary indices, and careful monitoring of prior settings in PSEM regularisation of parameter estimates (Asparouhov & Muthén, 2024; Dueber & Toland, 2023; Petras & Meiser, 2024). These considerations were applied in this study.

Based on burnout theory and empirical evidence presented under substantive considerations and advances in statistical methodology, we hypothesise as follows:

The CBI exhibits a hierarchical bifactor structure comprising three distinct, interpretable, and criterion-valid residual factors with minor cross-loadings and a congeneric general factor.

### 2.8. The Present Study

In this study, we sought to advance the measurement and theory of burnout by determining whether the data supported a CFA three-factor model, a second-order model, a two-factor, an essentially unidimensional model, or the hypothesised hierarchical PSEM bifactor congeneric model using student sample data.

The data for this study were collected in 2020/21 as part of a PhD multilevel research project during the COVID-19 pandemic, examining the crossover effects of lecturers’ and students’ engagement and well-being. The study employed eight measures, including the CBI and the SDRS. The PhD study used a smaller matched student–lecturer sample (N = 1594) after excluding non-matching cases (van der Ross, 2021). In contrast, this analysis included the full sample of 1938 students to explore a specific psychometric issue: the dimensionality of the 19-item CBI adapted for students using contemporary modelling techniques.

The use of existing datasets for secondary analysis is increasingly established, particularly when reported transparently and paired with distinct research questions and methodological techniques that advance theory (Cheng & Phillips, 2014; Kelly et al., 2024; Weston et al., 2019). This approach is gaining acceptance and aligns with the substantive-methodological synergy approach to assessing measurement models using advanced analytic techniques, yielding new insights while acknowledging potential biases (Asparouhov et al., 2015; Byrne et al., 2025; B. Muthén & Asparouhov, 2012).

van der Ross (2021) slightly adapted the CBI by rephrasing “work” as “academic work” in the work-burnout scale and “client” as “peers” in the client-burnout scale to make them relevant to students’ experiences of study burnout and peer-interaction burnout in an academic context. The CBI’s attributional structure has allowed it to be adapted to its context of use (e.g., reworded as patients in healthcare or colleagues and coursework in education) while preserving its structure, as demonstrated in previous studies (Campos et al., 2013; Thrush et al., 2021).

## 3. Materials and Methods

### 3.1. Study Design

We used a cross-sectional survey design to obtain quantitative data to assess the measurement model and relationships among variables. Cross-sectional data do not allow for testing causal relationships or the directionality of model paths. However, Kline (2015) argued that directionality may be a plausible assumption for regression paths when the rationale and supporting evidence are strong. In this study, we relied on prior research to support the directionality assumptions and inferences in the SEM model.

### 3.2. Study Sample

A purposive non-probability sampling strategy was used. Participants were recruited from undergraduate students enrolled at a South African higher education institution in 2020 and 2021. Data collection was conducted via an open electronic survey link available on the university’s online learning platform for all 5294 undergraduate students enrolled at the institution.

Electronic consent was obtained from all the participants. Of the 5294 students who consented, 3356 withdrew by exiting the survey, leaving a final sample of 1938 (36.6%). In response to the reviewers’ concerns regarding the high attrition rate and potential non-response bias, we expanded the manuscript to provide a more detailed account of the attrition pattern, its impact on the study’s demographics, and possible effects on response bias.

Significant attrition rates are not unusual in large-scale institutional surveys, typically 30–40%, and need not imply nonresponse bias when they are associated with sufficient sample representativeness and adequate statistical precision (Baruch & Holtom, 2008; Dillman et al., 2014). Wu et al. (2022) reported an average response rate of 44.1% across 1071 online educational research samples, highlighting the difficulty of achieving high participation rates.

Of the 3356 students who withdrew, 55.3% (1857) exited immediately after the consent section, before completing the demographic or substantive sections. Another 11.6% (389) completed the demographics but withdrew before the substantive items; 32.4% (1089) withdrew before 66% completion; and an additional 0.6% (21) withdrew before 82% completion.

To assess demographic-related attrition that may be associated with response bias, we compared the demographic distributions of the final 1938 students with those of the 1499 students who provided demographic information but did not complete the survey. Gender did not differ significantly (χ^2^(1) = 0.39, *p* = 0.531, Cramér’s V = 0.010). Significant differences were observed for language, χ^2^(11) = 26.07, *p* = 0.006, V = 0.085; ethnic group, χ^2^(4) = 29.79, *p* < 0.001, V = 0.091; and age, χ^2^(4) = 16.06, *p* = 0.003, V = 0.067. However, all effect sizes were small (V < 0.10), and the largest discrepancies ranged from 1.04 to 4.60 percentage points. These findings indicate limited demographic-related attrition among participants with available demographic information, although unmeasured reasons for withdrawal that affect response bias cannot be ruled out in this study.

Fosnacht et al. (2017) and Groves and Peytcheva (2008) emphasise that low response rates are not synonymous with nonresponse bias; bias depends on whether participation rate is related to substantive variables. The original survey (van der Ross, 2021) contained 119 substantive items; therefore, attrition may have reflected response burden, competing demands, lack of incentives, or insufficient motivation (Fan & Yan, 2010; Groves et al., 2009). Approximately two-thirds of those who withdrew did so before the substantive items, reducing the likelihood that the content directly prompted withdrawal, although construct-related withdrawal cannot be ruled out.

Nonresponse is likely more consequential for prevalence estimates, such as the proportion of students experiencing burnout, than for factor loadings, factor structure, reliability, and correlations. Nevertheless, these estimates are not immune to systematic exclusion related to substantive variables, particularly when attenuation is significant, which can alter the covariance matrix and affect the factor-analytic results. This remains an important area for further studies.

The sample included slightly more women (56.0%) than men (44.0%), indicating a moderately female-dominated sample. The age distribution showed that most respondents were in the 20–22-year age group (34.4%), followed by those aged 23–25 years (27.1%). Smaller proportions were aged 26–28 years (12.1%) and 17–19 years (9.1%), while 17.4% were classified as other. Overall, the sample was concentrated in the typical age range of undergraduate students.

The ethnic group distribution revealed that the sample was predominantly African (92.6%). Small minority groups included Coloured at 3.0%, Indian/Asian at 3.5%, with White at 0.1%, Other at 0.5%, and missing responses at 0.4%. This reflects a largely homogeneous ethnic profile, which aligns partly with South Africa’s 2022 population distribution: 81% African, 7.3% White, 8.2% Coloured, and 2.7% Indian (Statistics South Africa, 2023).

Sesotho was the dominant home language (45.8%), followed by English (19.7%), Setswana (12.7%), isiXhosa (9.7%), Afrikaans (4.4%), isiZulu (3.7%) and Xitsonga (1.1%). Smaller language groups included Tshivenda (1.0%), SiSwati (0.8%), Sepedi (0.7%), Other (0.2%), isiNdebele (0.2%) and missing responses (0.1%). The language profile was diverse, but centred mainly on Sesotho, English and Setswana.

### 3.3. Ethics Statement

The study was approved by the Faculty of Economic and Management Sciences Research Ethics Committee at the University of Pretoria, Pretoria, South Africa (Protocol EMS105/20), and was conducted in accordance with the Declaration of Helsinki and the Protection of Personal Information Act (POPIA). Participation was voluntary, with no penalty or loss of benefit for non-participation or withdrawal.

The introductory page of the survey instrument explained that the study investigated factors contributing to undergraduate student engagement and well-being, that the survey would take approximately 20 min to complete, and that responses would be treated confidentially and reported only in aggregated form. Participants were also informed that they could contact the researcher with any questions or comments regarding the study.

Participants consented to the study by clicking the survey link to begin, thereby indicating that they understood the study’s purpose and that their participation was voluntary. Survey responses were used for research purposes only, and the data were de-identified before statistical analyses and reporting to protect participants’ confidentiality.

### 3.4. Measures Used

#### 3.4.1. Copenhagen Burnout Inventory

Student burnout risk was assessed using the 19-item CBI on a five-point Likert scale, anchored at 1 = always/very high degree and 5 = never/very low degree (Kristensen et al., 2005). The wording of the items was slightly adapted to the study context by van der Ross (2021). The measure comprises three dimensions: personal burnout (six items), academic/study burnout (seven items), and peer burnout (six items). Sample items included “How often do you feel worn out?” for personal burnout, “Do your studies frustrate you?” for study-related burnout, and “Does it drain your energy to work with peers?” for peer burnout. Previous research has reported acceptable reliability for the CBI subscales, with Cronbach’s alpha coefficients of 0.85 for personal burnout, 0.87 for work burnout, and 0.82 for client burnout. See items in Supplementary Materials File S1.

#### 3.4.2. Study Resources Scale

Study resources were measured using the Study Resources subscale, comprising 18 items across four dimensions of the 23-item SDRS (Mokgele, 2014). van der Ross (2021) made minor adaptations to certain items to make the questions more module-specific rather than general to studies (e.g., “Do you receive sufficient information on the results of your studies?” was adapted to “Do you receive sufficient information on the results of your studies for the module?”). The Study Resources subscales were growth (six items), peer support (three items), lecturer support (five items), and information accessibility (four items). Items were rated on a four-point scale ranging from 1 = “never” to 4 = “always”. A sample item was “If necessary, can you ask your fellow students for help?” Previous research has reported reliability values above 0.70 for all dimensions of the study resource subscale. See items in Supplementary Materials File S2.

#### 3.4.3. Study Demands Scale

Study demands were measured using the Study Demands subscale of the SDRS (Mokgele, 2014). The scale comprises five items measuring time and study pressure. Items were rated on a four-point scale ranging from 1 = “never” to 4 = “always”. A sample item was “Do you have too much work to do in the module?” adapted to be module-specific by van der Ross (2021). Earlier work reported moderate reliability for the Study Demands Scale (ω = 0.61). In the present study, the one-factor Study Demands model showed acceptable reliability (ωS = 0.75). See items in Supplementary Materials File S2.

### 3.5. Descriptive Statistics

Descriptive statistics, such as the mean, skewness, and kurtosis, were employed to assess data distribution and normality. Influential outliers were detected using the Mahalanobis distance test, and the sensitivities of the chosen models were examined.

### 3.6. Analytical Strategy for the CBI Model Assessment

We viewed the Likert scale as rank-ordered rather than continuous. Accordingly, we conducted structural equation modelling (SEM) using Mplus 9.1 (L. K. Muthén & Muthén, 2026) employing weighted least squares mean and variance-adjusted (WLSMV) estimation with a sample of 1938 participants. The data contained no missing responses.

The analytic sample of 1938 was sufficient and exceeded the recommended 1000 for estimating complex latent-variable models for ordinal variables using WLSMV (Nussbeck et al., 2006). However, sample size adequacy in SEM also depends on model characteristics, parameter magnitudes, reliability, missingness, and relationships among variables. Rather than relying on sample size rules, adequacy was supported by model convergence, the absence of inadmissible solutions, and precise parameter estimates (e.g., small standard errors < 0.05) (Wolf et al., 2013). The large sample size reduced the usefulness of statistical significance in evaluating parameter salience; consequently, model interpretation emphasised the magnitude, precision, and theoretical relevance of the parameter estimates (Alamer, 2022).

We adopted a theory-guided, multi-stage analytical strategy, depicted in Figure 1, to evaluate the dimensionality and score interpretation of the CBI (Swami et al., 2023).

In the first stage, we tested the three-factor (3-factor CFA) and two-factor (2-factor CFA) CFA models reported in the literature, which served as the baseline models (see Figure 2). The three-factor CFA model represented the expected personal, study, and peer-related burnout dimensions. The two-factor CFA model was tested as a more parsimonious alternative, with expected personal and study burnout grouped as a single factor and peer-related burnout dimensions as the second factor. The three-factor PSEM model (3-factor PSEM^C^) retained the target factor structure while allowing minor cross-loadings through ALF priors to assess the prevalence of salient-factor cross-loadings. We then tested the Bifactor PSEM^C^ with three residual or specific factors and cross-loadings using the recommended zero-mean ALF priors with a variance of 1 to determine whether the CBI was best represented by a dominant general burnout factor, specific burnout factors, or both (see Figure 3) (Asparouhov & Muthén, 2024). Furthermore, we modelled an alternative bifactor solution in which personal burnout was not regarded as a separate factor but was combined with study burnout. This approach aimed to evaluate whether its inclusion was substantively necessary or if merging personal and study burnout items into a single residual factor offered a more coherent representation of the data. The prior constraint was monitored for suitability using the ALF penalty ratio, which should typically fall between 0.01 and 0.10.

In the second stage, we estimated a bifactor PSEM^CR^ model with all cross-loadings and correlated residuals specified using the ALF (0,1) prior to identify the main sources of model misspecification. Consistent with Bayesian SEM, this approach distinguished minor misspecifications or white noise from substantive misspecifications (Asparouhov et al., 2015). Subsequently, we balanced parsimony and fidelity by constraining all correlated residuals to zero and freeing only those that were substantively important and theoretically defensible, resulting in a parsimonious, theoretically supported bifactor PSEM^CR−1^ model with one correlated residual freed (Figure 3). Subsequently, we evaluated a one-factor confirmatory factor analysis (CFA) model (1-factor CFA-1), which incorporates the freely estimated correlated residual, to assess the relative bias of the factor compared to the general factor within the bifactor model. This analysis aimed to determine whether the assumption of an essentially unidimensional model is justified for the CBI when researchers intend to use only a single unit-weighted score in subsequent analyses (Dueber, 2017).

### 3.7. Analytical Strategy for Assessing the Criterion Measures

The criterion measures were analysed using a bifactor PSEM model for the Study Resources Scale, with cross-loadings estimated using ALF priors and a one-factor CFA model for the Study Demands Scale. The general and specific factors were correlated with the CBI factors, as illustrated in Figure 4. We derived plausible values (PVs) from the measurement models using the missing data imputation utility in Mplus and the Bayes estimator (Asparouhov & Muthén, 2010). We used 10,000 iterations to impute 30 PV datasets, and convergence was monitored using a potential scale reduction (PSR) indicator (PSR should be below 1.05) and trace plots. For all estimates, we adopted the MPlus default settings and combined the PV datasets using Rubin’s (1987) method. These PVs, representing latent variables, were saved for the selected CBI, Study Resources, and Study Demands models and used in the secondary regression analyses. We used plausible values rather than including full measurement models in the regression to preserve the selected measurement structures, correct for factor score unreliability, and avoid interference between the measurement and structural parts of the analysis (Asparouhov & Muthén, 2010; Rosseel, 2020).

### 3.8. Model Assessment Criteria

Model evaluation was based on global fit, local misspecification diagnostics, factor-loading patterns, correlated residuals, factor strength indices, and theoretical interpretability. We used CFI, TLI, and RMSEA to evaluate overall fit, following the conventional cut-offs of CFI ≥ 0.90, TLI ≥ 0.90, and RMSEA ≤ 0.08. We relied on changes greater than 0.01 for CFI and TLI, and 0.015 for RMSEA, as meaningful differences in model fit (Chen, 2007). Local misspecification was examined using factor loadings, cross-loadings, correlated residuals, item, and factor bias (Asparouhov et al., 2015; Morin et al., 2016; Saris et al., 2009). Primary loadings of approximately λ ≥ 0.40 were treated as substantively meaningful, whereas cross-loadings below|0.20| were interpreted as minor unless they showed a clear pattern across conceptually related items. Correlated residuals were considered only when they were statistically and substantively defensible and supported by earlier CBI studies; for example, when similarly worded items, overlapping item stems, or shared content domains suggested local dependence.

For the bifactor PSEM model, we computed ω, ωH, ωS, ωHS, ECV, ECVss, PUC, FD, H, ARPB, VAR, and SS_λ_ using the Dueber (2017) bifactor indices calculator. To assess the degree of essential undimensionality underlying the bifactor model’s multidimensionality, we examined ECV, PUC, and ωH. PUC moderates the interpretation of ECV, which is interpreted alongside PUC rather than as an isolated index (Reise et al., 2013; Rodriguez et al., 2016b). In other words, is the multidimensional data unidimensional enough to support a unidimensional SEM model? We used the thresholds: ECV > 0.50 if PUC ≥ 0.80; ECV > 0.60 if PUC < 0.80; and ωH ≥ 0.70 to determine essential unidimensionality (Reise et al., 2013; Rodriguez et al., 2016b). We used ARPB values below 0.10–0.15 to indicate limited factor-score bias when forcing a unidimensional model on the data. In addition, we relied on FD ≥ 0.90 and H ≥ 0.70 to support the reliability of the optimal-weighted scoring of the general factor (i.e., congeneric models) (Dueber, 2017). For the residual or specific factors, we used the Dueber and Toland (2023) value-added criteria of VAR ≥ 1.10 on the ωS-moderated interpretation of ωHS and ECVss. The VAR ≥ 1.10 threshold values that apply for a bifactor model with three subfactors are presented in the Supplementary Materials Table S3. We also used the Petras and Meiser (2024) criterion of SS_λ_ ≥ 1.00 to evaluate whether the residual-specific factors were sufficiently stable and generalisable. An interpretable and stable specific factor had to meet both the VAR ≥ 1.10 and SS_λ_ ≥ 1.00 thresholds to be considered useful for inclusion in the criterion-validity analyses.

## 4. Results

### 4.1. Item Descriptive Statistics

For the CBI scale, descriptive statistics indicated a mean item score of 3.27 (range: 2.73–3.91) with a mean standard deviation of 1.20 (range: 0.95–1.37). The average skewness was −0.20 (range: −0.85 to 0.21), and the average kurtosis was −0.63 (range: −1.07 to 0.15). Mardia’s multivariate kurtosis statistic (50.72; *p* < 0.05) suggested a non-normal distribution. Additionally, no missing values were observed.

The SDRS had an average item score of 3.24 (range: 2.38–3.57), with a standard deviation of 0.75 (range: 0.54–1.23). The mean skewness was −1.04, with a range of −1.78 to 0.11, and the mean kurtosis was 0.46, with a range of −1.34 to 2.54. This suggests a non-normal distribution. No missing data were found.

The items for the CBI and SDRS are listed in Supplementary Materials Files S1 and S2, respectively. Influential outliers, as identified by the Mahalanobis distance test, had negligible impact on the fit statistics and parameter estimates of the selected bifactor PSEM models and were included in the analyses. All models converged successfully with no inadmissible solutions. The selected SEM measurement parameters (λ) and structural paths (β) demonstrated high precision, yielding standardised standard errors predominantly between 0.01 and 0.03 (none exceeding 0.05). Based on asymptotic normal theory, Wald Z-tests at this level of precision afforded adequate power (>0.80) to detect small standardized population effects in the 0.10–0.15 range—well below the a priori threshold for minor effects (<0.20).

### 4.2. Baseline CFA and PSEM Models

The results of the factor models examined are shown in Table 1. We first tested the baseline CFA and PSEM models for the Copenhagen Burnout Inventory (CBI). The three-factor CFA model showed a good fit: χ^2^ (149) = 1793.91, RMSEA = 0.075, CFI = 0.966, TLI = 0.961. The second-order three-factor model produced the same fit: χ^2^(149) = 1793.91, RMSEA = 0.075, CFI = 0.966, TLI = 0.961. The two-factor CFA model demonstrated a less-than-optimal fit, χ^2^(171) = 3050.73, RMSEA = 0.100, CFI = 0.940, TLI = 0.932, suggesting that items related to study and personal burnout are not homogeneous and should be modelled separately. The three-factor PSEM model with cross-loadings had a meaningfully reduced model fit: χ^2^(117) = 3251.69, RMSEA = 0.115, CFI = 0.935, TLI = 0.906.

Table 2 summarises the structures of the 3-factor CFA, alternative 2-factor^alt^ CFA, and PSEM models, including cross-loadings. Appendix A, Table A1 presents the factor structure and all factor loadings for the 3-factor CFA, 2-factor^alt^ CFA, and PSEM models, with cross-loadings.

The three-factor CFA model showed high mean primary loadings for personal burnout (F1: M_λ_ = 0.77), study burnout (F2: M_λ_ = 0.82), and peer burnout (F3: M_λ_ = 0.75). However, F1 and F2 were strongly correlated (r = 0.78), suggesting substantial common variance. F3 correlated modestly with F1 (r = 0.31) and F2 (r = 0.36), indicating that these factors are more distinct. In the second-order model, F1 (λ = 0.82) and F2 (λ = 0.95) contributed strongly to the general factor, whereas F3 (λ = 0.38) contributed modestly, suggesting that the common factor was mainly defined by personal and study-related burnout. Campos et al. (2013) found remarkably similar second-order loadings for related student burnout factors: personal-related burnout (λ = 0.86), study burnout (λ = 0.97), and peer burnout (λ = 0.39), suggesting structural generalizability.

The correlation between F1 and F2 in the present study is corroborated (r = 0.79; N = 34,727) in a large occupational study (Kováč & Lisá, 2025); however, client burnout factor F3 correlated more strongly with F1 (r = 0.58) and F2 (r = 0.71). Two studies on the student version of the CBI show that the F1/F2 correlation was high (e.g., r = 0.87; 0.88). However, the correlations between the peer burnout factor F3 and F1 and F2 (e.g., r = 0.35/0.35; 0.32/0.38) were modest (Bolatov et al., 2021; Campos et al., 2013), which aligns well with the present study. Similar to earlier studies, the reverse-scored item B13, “have enough energy for family and friends during leisure time,” showed a low factor loading (λ = 0.36) on F2 (Campos et al., 2013; Tran et al., 2023; Živanović et al., 2021). This shows that the CFI structure is largely preserved after adaptation for the current study and is generalisable.

The three-factor PSEM model with ALF priors reduced the mean primary loadings to 0.68 for F1, 0.78 for F2, and 0.69 for F3, respectively. The cross-loadings were generally small, with mean cross-loadings of 0.09, 0.07, and 0.00 for F1, F2, and F3. The largest cross-loading was 0.29. Compared with the CFA model, the ARPB values for the factor loadings were 0.17, 0.14, and 0.01, respectively, suggesting that the CFA model inflated the loadings for F1 and F2, whereas F3 was largely unaffected. The PSEM factor correlations were lower than those in the CFA model, particularly between F1 and F2 (r = 0.69), but remained sufficiently high to support further bifactor modelling. Overall, these findings support the model fit paradox (Wang et al., 2025): The IC CFA model fit was better than the PSEM model with cross-loadings, even though substantial mis-specified cross-loadings were present, which the CFA model absorbed and inflated F1 and F2.

### 4.3. Bifactor PSEM Model Analysis

The CBI bifactor PSEM^C^ model with cross-loadings improved the model fit meaningfully (Δ CFI/TLI ≥ 0.01, Δ RMSEA ≥ 0.015): χ^2^(101) = 1631.77, RMSEA = 0.086, CFI = 0.968, TLI = 0.947, although the RMSEA remained suboptimal (Table 1). The model (Table 3 and Appendix A, Table A2) showed a clearly defined general burnout factor (Gf; M_λ_ = 0.55; range = 0.25–0.90), with F1 (M_λ_ = 0.48; range = 0.36–0.60) and F3 (M_λ_ = 0.75; range = 0.64–0.83) also being clearly defined. In contrast, F2 was weakly defined (M_λ_ = −0.13; range = −0.45–0.12), with six significant cross-loadings for items B1, B2, B14, B15, B18, and B19, which ranged from −0.29 to 0.20.

The CBI bifactor^alt^ PSEM^C^ model with cross-loadings produced a significantly weaker fit (Δ CFI/TLI ≥ 0.01, Δ RMSEA ≥ 0.015): χ^2^ (117) = 3250.77, RMSEA = 0.116, CFI = 0.935, TLI = 0.906 (Table 1), confirming the residual specificity and indispensability of the personal burnout factor. The model (see Appendix A, Table A2) revealed a distinct general burnout factor (Gf; M_λ_ = 0.56; range = 0.25–0.89). The F1^alt^ (interburn) factor was overall weak (M_λ_ = 0.22; range = −0.09–0.60). However, the personal burnout items’ loadings on the factor were good (M_λ_ = 0.47; range = 0.34–0.60), and study burnout showed near-zero loadings (M_λ_ = 0.01; range = −0.09–0.10), suggesting that these two residual factors are not uniform.

To identify the main local misspecifications, a bifactor PSEM model was estimated with all cross-loadings and ALF (0,1)-regulated correlated residuals. Meaningful correlated residuals emerged (Figure 5) for B1/B2, B14/B15, and B18/B19, ranging from r = 0.45 to 0.49, with B1/B2 being the strongest and most commonly reported correlated residual in earlier studies (Maroco & Campos, 2012; Tran et al., 2023; Wongtrakul et al., 2021). Grouping the same-factor items together in the CBI, in addition to using parallel wording and similar content, may have led to response sets and elevated local dependencies (Loiacono & Wilson, 2020).

The impact of these residual correlations was assessed by comparing the bifactor PSEM^C^ model (Appendix A, Table A2), in which the residual correlations were constrained to zero, with the bifactor PSEM^CR^ model (Appendix A, Table A3), in which they were ALF-regulated. Although F2 remained weakly defined in the PSEM^CR^ model (M_λ_ = 0.01; range = −0.37–0.34), all cross-loadings were minor, ranging from −0.05 to 0.08. This suggests that the F2 cross-loadings in the bifactor PSEM^C^ model largely reflect the residual item dependencies. Guided by theory and empirical evidence, correlated residuals were therefore freed conservatively, and model independence was preserved by freeing only the theoretically defensible B1/B2 residual found in earlier studies. The bifactor PSEM^CR−1^ model with cross-loadings showed a further meaningful improvement in model fit, suggesting a good fit: χ^2^(100) = 915.39, RMSEA = 0.063, CFI = 0.983, TLI = 0.971 (See Table 1). The prior proportion was 0.06, which is an acceptable penalty ratio for the selected ALF (0,1) prior.

As shown in Summary Table 3, constraining all residual correlations to zero, except for B1/B2, increased ARPB by small amounts (1%, 6%, 11%, and 6% for Gf, F1, F2, and F3, respectively; ARPB ≤ 0.10–0.15). The effects on ECV, ECVss, and ωHS were similarly minor and inconsequential. Thus, retaining constraints on the three additional residual correlations had a negligible impact on the factor structure while preserving most of the model’s independence and degrees of freedom. Accordingly, only the selected bifactor PSEM^CR−1^ model’s factor structure and auxiliary indices are discussed further.

The bifactor PSEM^CR−1^ model showed a clearly defined general burnout factor (Gf), with mean loadings of 0.56 (range: 0.26–0.86). Gf showed high reliability (ωS = 0.95), strong factor determinacy (FD = 0.97), high construct replicability (H = 0.94), and near-threshold omega hierarchical (ωH = 0.77). However, ECV = 0.58 with PUC = 0.70, requiring interpretation against the higher approximate ECV threshold of 0.60. Thus, Gf did not fully meet the criteria for essential unidimensionality, indicating a dominant but not exclusive general burnout factor. The heterogeneous loadings further suggest a congeneric measurement structure that favours optimal-weighted scoring (McNeish & Wolf, 2020). In contrast, the strict one-factor model showed poor fit and substantial parameter bias relative to the bifactor Gf (ARPB = 0.54), supporting the presence of meaningful residual factors, particularly F1 and F3.

The specific factors differed markedly in terms of added value. Factor F1 showed moderate loadings (M_λ_ = 0.44; range = 0.32–0.54), acceptable reliability (ωS = 0.89), and sufficient factor strength (SS_λ_ = 1.22). Its ECVss = 0.36 and ωHS = 0.31 indicated interpretable residual specificity and added value beyond Gf. Factor F2 showed negligible residual specificity, with M_λ_ = 0.02, ECVss = 0.09, ωHS = 0.00, and SS_λ_ = 0.39. Therefore, it failed to meet the criteria for interpretability or added value and was excluded from the criterion-validity analyses. F3 showed the strongest residual specificity and added value, with high loadings (M_λ_ = 0.75; range = 0.64–0.85), acceptable reliability (ωS = 0.89), substantial residual common variance (ECVss = 0.83), good residual subscale reliability (ωHS = 0.72), and sufficient factor strength (SS_λ_ = 3.40). These results support F3 as a meaningful specific factor beyond general burnout.

Overall, the bifactor PSEM^CR−1^ model was retained as the final CBI model. The results supported a dominant general burnout factor, with a distinct, stable, and interpretable residual-specific peer burnout factor. Personal burnout also met the residual-specificity criterion, albeit to a lesser extent. The study burnout factor was central and strongly embedded in the general factor, with homogeneous, high-factor marker loadings, leaving insufficient residual specificity after controlling for the general factor. Therefore, the internal structure of the CBI bifactor model supports a dominant and stable general factor, along with two stable, statistically interpretable residual factors that add value beyond the general factor. The assessment results of the criterion validity of the bifactor model are as follows:

### 4.4. Criterion-Measure Models

Next, we assessed the Study Resources and Study Demands scales as criterion measures (See Table 4). The one-factor CFA model for study demands, with one correlated residual, showed an acceptable fit (χ ^2^(4) = 47.66, RMSEA = 0.075, CFI = 0.983, TLI = 0.959). The study demands factor showed moderate-to-high loadings (M_λ_ = 0.68; range = 0.38–0.75) and acceptable reliability (ωS = 0.75) (See Table 5 for summary and Supplementary Materials Table S4 for the factor loadings matrix).

The bifactor PSEM model for study resources also showed a good fit, χ^2^(73) = 287.75, RMSEA = 0.036, CFI = 0.991, TLI = 0.980. The general resources factor showed moderate loadings (M_λ_ = 0.56; range = 0.10–0.84), high reliability (ωS = 0.93), strong factor determinacy (FD = 0.95), and high factor replicability (H = 0.93). The indices PUC = 0.78, ECV = 0.65, and ωH = 0.83 supported a unit-weighted scorable essentially unidimensional general resources factor (See Table 5 for a summary and Supplementary Materials Table S4 for the factor loadings matrix).

The residual factors showed varying levels of added value. Growth (F1) showed moderate loadings (M_λ_ = 0.38), acceptable reliability (ωS = 0.84), interpretability (ECVss = 0.37, ωHS = 0.30), and borderline stability (SS_λ_ = 1.00), indicating acceptable added value. Peer support (F2) showed the greatest added value, with strong loadings (M_λ_ = 0.74) and acceptable reliability, stability, and interpretability (ωS = 0.84, ECVss = 0.86, ωHS = 0.72, and SS_λ_ = 1.67). Lecturer support (F3) showed minor value-added, with ECVss = 0.19 and ωHS = 0.12, but its SS_λ_ = 0.55 did not support a stable factor-score interpretation. Information accessibility (F4) did not meet the required criteria, with weak loadings (M_λ_ = 0.21) and low added value and interpretability (ECVss = 0.11, ωHS = 0.09, and SS_λ_ = 0.18). Consequently, only the general resources factor, growth, and peer support were retained for the regression analyses to determine the criterion validity of the CBI.

### 4.5. Criterion-Validity Regression Analyses

We used plausible values to represent latent variables or true scores from the selected CBI, Study Resources, and Study Demands models in the regression analyses (See Table 6 and Table 7). The regression model was saturated (χ^2^(0) = 0.00, RMSEA = 0.00, CFI = 1.00, TLI = 1.00); therefore, model fit was not informative, and interpretation focused on the regression paths corrected for score unreliability.

The study found that general burnout was positively predicted by study demands (β = 0.37, SE = 0.03, *p* < 0.001), whereas the study resources general factor negatively predicted general burnout (β = −0.34, SE = 0.03, *p* < 0.001). Growth also showed a small negative effect on general burnout (β = −0.10, SE = 0.04, *p* < 0.001), whereas peer support was not significant (β = 0.02, SE = 0.02, *p* = 0.35). Together, the criterion variables explained 32% of the variance in general burnout, supporting the study demands and resources theory (Bakker & Mostert, 2024; Jagodics & Szabó, 2022; Mokgele & Rothmann, 2014).

The personal burnout residual factor was not significantly predicted by any of the criterion variables. Study demands, general study resources, growth, and peer support all showed non-significant paths, and the explained variance was negligible (R^2^ = 0.00, *p* = 0.31). These findings suggest that personal burnout does not add meaningful criterion-related validity beyond the general burnout factor.

Peer burnout showed clear criterion-related distinctiveness. Study demands (β = −0.13, SE = 0.03, *p* < 0.001), general study resources (β = −0.18, SE = 0.03, *p* < 0.001), growth (β = 0.10, SE = 0.03, *p* < 0.001), and peer support (β = −0.50, SE = 0.02, *p* < 0.001) were significant predictors. The strongest effect was found for peer support, indicating that higher peer support was predominantly associated with lower peer burnout, followed by general study resources, which were also associated with lower peer burnout and were supported by theory (Bakker & Demerouti, 2017; Jagodics & Szabó, 2022; Mokgele & Rothmann, 2014). The small but statistically significant negative association between study demands and peer burnout (β = −0.13) and the small positive association between growth and peer burnout (β = 0.10) are counterintuitive, lack a theoretical basis, and may be sample-specific; therefore, they are not interpreted further. Overall, the criterion variables explained 29% of the variance in peer burnout.

The residual correlation between personal and general burnout was negative and significant (r = −0.15, SE = 0.03, *p* < 0.001), whereas the residual correlations of peer burnout with general burnout and peer burnout with personal burnout were not significant. Overall, the regression results supported the criterion validity of the general burnout factor and provided evidence for the added value of peer burnout. In contrast, personal burnout added little value to the criterion model. The criterion validity evidence supports a CBI bifactor PSEM model, with a dominant general factor and one stable, statistically interpretable residual factor that adds value beyond the general factor. More nomological evidence is needed to support the uniqueness of the personal burnout residual factor over the general factor.

## 5. Discussion

This study addressed the need to re-examine the CBI’s dimensionality beyond the previously reported three-, two-, and second-order CFA models by testing these conventional alternatives before estimating a bifactor PSEM model. Although the three-factor CFA and second-order models showed acceptable global fit, subsequent PSEM results with cross-loadings indicated inflated factor loadings and factor correlations. The significant shared variance among the CFA factors further called the subscales’ distinctiveness into question. These findings show that global fit indices and salient factor loadings, interpreted at face value, are insufficiently sensitive to identify substantive model misspecifications or justify distinct subscale interpretations. Therefore, drawing conclusions from subscale scores that lack discriminant validity is questionable.

Utilising bifactor auxiliary indices, PSEM with ALF priors, and global fit indices, we observed only partial support for the hypothesised CBI, which demonstrates a hierarchical bifactor structure comprising three distinct, interpretable, and criterion-valid residual factors with minor cross-loadings and a congeneric general factor. More specifically, a dominant congeneric general burnout factor emerged, along with two distinct interpretable residual factors: personal burnout and peer burnout. Only the general burnout factor and peer burnout demonstrated criterion validity with the SDRS. Personal and study burnout shared substantial common variance with the general factor, whereas peer burnout was more clearly differentiated (Moser et al., 2023; Rocha et al., 2020; Živanović et al., 2021). Study burnout was largely absorbed by the general factor, followed by personal burnout, which showed interpretable residual factor specificity (ECVss = 36%); however, it requires criterion validation.

### 5.1. Substantive Findings and Insights

The selected bifactor PSEM model with ALF priors for cross-loadings and vetted free-correlated residual supports the presence of a predominant general burnout factor; however, it does not represent a strictly unidimensional interpretation, as evidenced by the ARPB index. The general factor demonstrated reliability and determinacy, accounting for the dominant proportion of shared variance (ECVss = 58%) across all items. Nonetheless, the lower ECV, PUC, and ωH < 0.80, along with heterogeneous factor loadings, indicate that the CBI should not be regarded as essentially unidimensional, thereby allowing unit-weighted scoring. Instead, the findings suggest a congeneric general burnout factor that warrants careful scoring and interpretation, preferably with an optimally weighted scoring approach (McNeish & Wolf, 2020; Rodriguez et al., 2016b). A critique of optimal-weighted scoring models is their limited generalisability when applied to smaller sample sizes (Liu & Pek, 2023). However, this is a large sample, and the correlational pattern among the CFA factors in the study was similar to that in other studies, suggesting that the heterogeneous factor-loading pattern and the congeneric general factor are likely applicable to other samples as well (Moser et al., 2023; Rocha et al., 2020; Živanović et al., 2021).

The general factor identified here should not be interpreted as a theoretical axiom of the CBI, but neither is it theoretically disconnected from its original conceptualisation. Exhaustion has long been regarded as central to burnout theory, including the MBI tradition and, more recently, the BAT (Aguayo-Estremera et al., 2024; De Beer et al., 2026), while Kristensen et al. (2005) explicitly defined physical, emotional, and mental exhaustion as the core of burnout. The distinctive feature of the CBI is therefore not that its three domains represent fundamentally different burnout phenomena, but that the same underlying exhaustion is attributed to increasingly specific contexts—personal, work, or study, and peer. This provides a substantive basis for expecting considerable common variance alongside meaningful domain-specific variance. Consistent with this interpretation, the broader CBI literature reviewed in this study repeatedly demonstrates substantial overlap among its dimensions, with some studies supporting a hierarchical model (Maroco & Campos, 2012; Rocha et al., 2020). Thus, the current bifactor findings should be seen as an empirical representation of a theoretically plausible common exhaustion core with attributional specificity. However, the strength and precise structure of that general factor remain empirical questions that require replication. Therefore, interpretations of the general burnout factor should be approached with caution until further studies are conducted. Future research might further refine the overall ‘burnout’ construct into its components or subtypes.

The general burnout factor exhibited criterion validity, demonstrating a medium effect size relationship with the Study Demands factor and the general factor within the bifactor Study Resources scale. Note that PVs as latent variables were employed in the criterion-validity analysis to address unreliability among interrelated variables, thereby ensuring greater accuracy in the findings (Rhemtulla & Savalei, 2025). These results imply that, consistent with theoretical expectations, students’ overall burnout risk increases under demanding academic conditions and decreases when general resources are available. The imbalance caused by insufficient resources to meet these demands aligns with burnout theory (Bakker & Mostert, 2024; Jagodics & Szabó, 2022; Mokgele & Rothmann, 2014). Notably, the strength of the relationships between the variables did not differ significantly (r = 0.37 for Study Demands and r = −0.34 for the general Study Resources factor), indicating a balanced relationship within the sample. Had any of these correlations been notably higher, the assumption of balance in the burnout theory might have been less well-supported in this study. The deeply embedded study burnout construct in the general factor is directly tied to study demands, such as workload and deadlines, and leads to academic exhaustion. However, study burnout is also tied to study resources, growth, peer support, lecturer support, and information accessibility, which, in turn, diminishes exhaustion (Jagodics & Szabó, 2022; Mokgele & Rothmann, 2014). The imbalance between study demands and resources determines the level of exhaustion and burnout a person experiences. Growth, lecturer support, and information accessibility are critical in this relationship and are best represented by the study resources’ general factor in the bifactor model, whereas peer support is less well embedded, with greater residual factor specificity (van Zyl et al., 2024). The following paragraph explores the distinctive relationship between peer support and peer burnout, then transitions to personal burnout.

Peer burnout was distinctly characterised by substantial residual factor specificity (ECVss = 0.83), high factor determinacy, and significant criterion-related validity. The pronounced negative correlation between peer support and peer burnout (r = −0.50) indicates that peer-related exhaustion is not merely another manifestation of general burnout, but a relationally specific dimension of students’ academic experience. This finding is theoretically important because interpersonal exhaustion in academic peer contexts may constitute a distinct component of student burnout rather than the core burnout syndrome, particularly where group work, peer dependence, and collaborative learning are prevalent (Maroco & Campos, 2012). The negative association between the general resources factor, which included peer support, and peer burnout further suggests that peer burnout depends substantially on the quality of peer interaction. According to Maroco and Campos (2012), encouraging and motivational relationships with fellow students may counteract peer burnout. More specifically, peer support entails providing emotional, informational, and practical support in a reciprocal, mutually beneficial manner (van Zyl et al., 2024). Conversely, when this balance is compromised, as reflected in the CBI item “you give more than you get back when you work with peers,” peer burnout becomes more evident. Overall, the evidence suggests that peer burnout among students is moderately associated with peer support and should be considered a distinct or unique contributor to burnout in academic environments (Maroco & Campos, 2012; Mokgele & Rothmann, 2014).

Personal burnout is slightly less prominent in the general burnout factor, not solely attributable to work, forming a minor yet stable and interpretable residual factor. This residual factor accounts for 36% of the within-factor shared item variance and is interpreted as non-work-related burnout within the framework of the theory (Kristensen et al., 2005), supported by near-zero correlations with the study demands and study resources factors. Personal burnout, distinct from study burnout, addresses the broader question of how tired and exhausted a person is, which is attributable to multiple causes (e.g., work, health, family demands, other life circumstances) rather than to specific causes (Kristensen et al., 2005). This residual factor in the bifactor model offers a valuable opportunity for future research to explore the true nature of personal burnout free from the influence of shared variance with student burnout.

Not only do bifactor models and PSEM with ALF priors provide a unique opportunity to study constructs where the sources of variance have been clearly differentiated, but they also allow for interpretations of differentiated scores between shared and unique reliable variance. A general-plus-specific scoring approach is recommended. In this context, the general factor score serves as a broad indicator of burnout, whereas stable and interpretable residual factors can be more diagnostic and nuanced (DeMars, 2013; Schaap & Botha, 2026). Moreover, the relationships between the CBI and SDRS identified in this study enrich the interpretation of scores.

For example, someone might have a moderate overall burnout score but a high personal burnout residual score, indicating more pronounced personal burnout symptoms than those related to study or peer burnout. This suggests that burnout involves more than study demands and resources, and that it requires interventions beyond the study environment. Conversely, an individual could score high on general burnout but low on the peer and personal burnout residual factors, suggesting that they experience high study demands and limited institutional support, yet enjoy strong peer backing, according to the SDRS bifactor model. In such cases, burnout tends to be attributional or situational (Kristensen et al., 2005), potentially requiring personalised or institutional intervention. Addressing this at an individual level—by assisting the person in managing study demands and optimising institutional resources, such as lecturer support and information resources, which are key components of the general factor in the SDRS bifactor model—may effectively reduce study burnout, which is integral to general burnout in the CBI bifactor model.

### 5.2. Methodological Findings and Insights

In this study, we adopted a substantive methodology synergy approach to bridge the divide between innovations in contemporary statistical analysis techniques published in methodological journals and their adoption in applied research published in substantive-focused journals (Hofmans et al., 2021; Marsh & Hau, 2007).

This study demonstrated that a good CFA global fit does not necessarily support a plausible three-factor CBI model, even though this model has frequently been reported as the preferred model in previous research (Johnson, 2024; Kováč & Lisá, 2025; Montgomery et al., 2021; Thrush et al., 2021). The use of modern statistical techniques to identify and diagnose model misfits not captured by global fit indices was not evident in the validation studies discussed earlier. Rodriguez et al. (2016a) clearly substantiated why all correlated multidimensional self-report psychological measures should be scrutinised using bifactor models with auxiliary indices as a psychometric tool to differentiate the sources of variance in items, even when the proposed theoretical model is not hierarchical.

Only after testing a sequence of models and applying more contemporary bifactor PSEM analytical techniques and auxiliary indices did we uncover the CFA model fit paradox for the CBI, as noted earlier. The model showed good CFA global fit (CFI = 0.97, TLI = 0.96, RMSEA = 0.08) and did not reveal substantively mis-specified cross-loadings or correlated residuals that inflated factor loadings and correlations. More specifically, the illusion of a well-defined three-factor CFA model was revealed through bifactor indices, indicating that most of the variance is attributable to a common burnout continuum, as noted earlier. Moreover, local misspecification analysis with PSEM and ALF priors revealed substantive misspecifications in the model, including cross-loadings and correlated residuals. Rather than relying on sequential, data-driven freeing of residuals, this study allowed cross-loadings and correlated residuals to be estimated concurrently within an identified PSEM model and controlled with ALF priors, clearly differentiating minor and important misspecifications (Asparouhov & Muthén, 2024). We resolved all misspecifications and retained only those that were theoretically and empirically defensible in a more independent CBI bifactor model.

The use of plausible values in the criterion-validity regressions strengthened the analysis by preserving the selected measurement models and enabling a more accurate examination of the relationships among latent variables without introducing unnecessary measurement-structural interference.

### 5.3. Limitations

Several limitations should be considered. First, the study used cross-sectional survey data, which precluded causal inference from the regression paths. Although the direction of associations was theoretically grounded in the study demands-resources framework, longitudinal designs are needed to examine whether demands and resources predict subsequent burnout or whether burnout changes students’ perceptions of demands and resources. Second, the data were collected from one South African higher education institution during the 2020/2021 COVID-19 period. Therefore, the findings may reflect sample-specific institutional, cultural, and pandemic-related conditions and should be replicated at other universities and in other countries, as well as among post-pandemic student cohorts.

Third, the sample was obtained through non-probability sampling and was demographically uneven, with a predominantly African and Sesotho-speaking profile. This limits generalisability and highlights the need for measurement invariance testing across language, gender, ethnicity, and institutional groups. Fourth, the 64% attrition rate among students who completed the survey may have introduced response bias. Fifth, the use of a self-report instrument and a single-source cross-sectional design introduces the possibility of common method variance and self-report bias (Podsakoff et al., 2012). However, different scale formats and anchors across the measures mitigate this concern (Podsakoff et al., 2012).

Sixth, the study used secondary data originally collected for a broader research purpose. Although the secondary analysis was justified by a new, very specific psychometric research question and more advanced methods, the study was constrained by the available measures and design. The strong findings for peer burnout are promising; however, future studies should examine how the factor behaves across different forms of academic peer interaction, such as group work, informal peer support, and collaborative learning.

### 5.4. Conclusions

This study advances the interpretation of the adapted CBI by showing that student burnout may be best represented by a dominant, yet not strictly unidimensional, congeneric general burnout factor, within a bifactor PSEM framework. Although this general factor should not be regarded as a theoretical axiom of the CBI, its emergence is theoretically plausible, given the common exhaustion core, and warrants further refinement and replication studies. Study-related burnout failed to demonstrate meaningful residual specificity, personal burnout retained modest specificity, and peer burnout demonstrated clear specificity and significant added value. These findings caution against the routine interpretation of all three CBI subscales as distinct and suggest that further studies relying solely on IC-CFA may overlook important shared variance and local model misspecifications. For research and practice, the general burnout factor may therefore, with caution, serve as the primary indicator of student burnout, while peer burnout can provide additional insight into relationally specific exhaustion in academic settings. More broadly, the study shows how substantive burnout theory and advanced psychometric modelling can be integrated to produce more defensible measurement, scoring, and interpretation decisions.

## Figures and Tables

**Figure 1:**
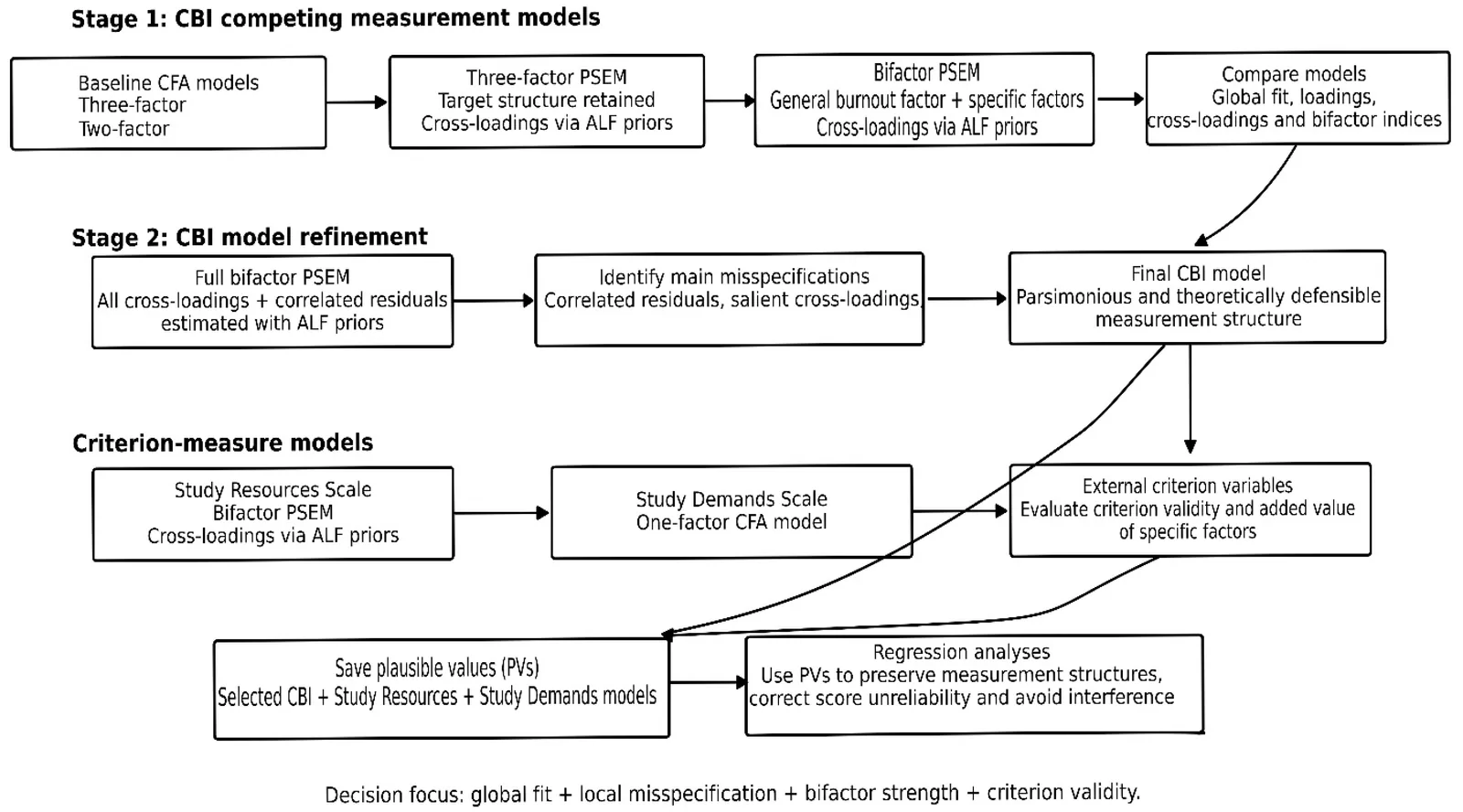
Sequential analytical strategy for CBI model assessment and criterion-validation analyses.

**Figure 2:**
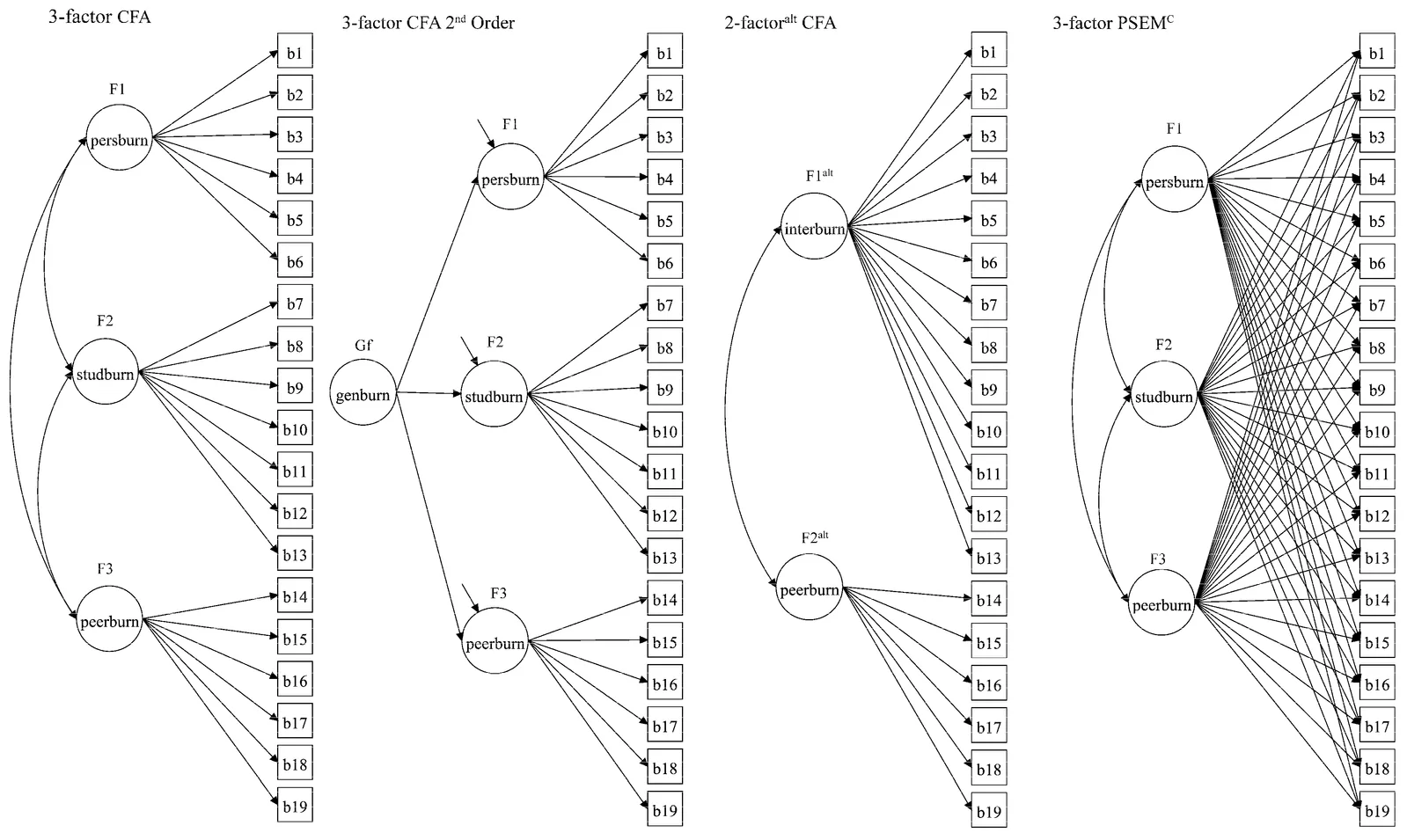
Models Tested, Notes: genburn (Gf), = General burnout factor; persburn (F1) = Personal burnout; studburn (F2) = Study burnout; peerburn (F3) = Peer burnout; interburn (F1^alt^) = persburn and studburn factors combined in the alternative model; peerburn (F2^alt^) = Peer burnout in the alternative model; ^alt^ = Alternative model; ^C^ = with item cross-loadings.

**Figure 3:**
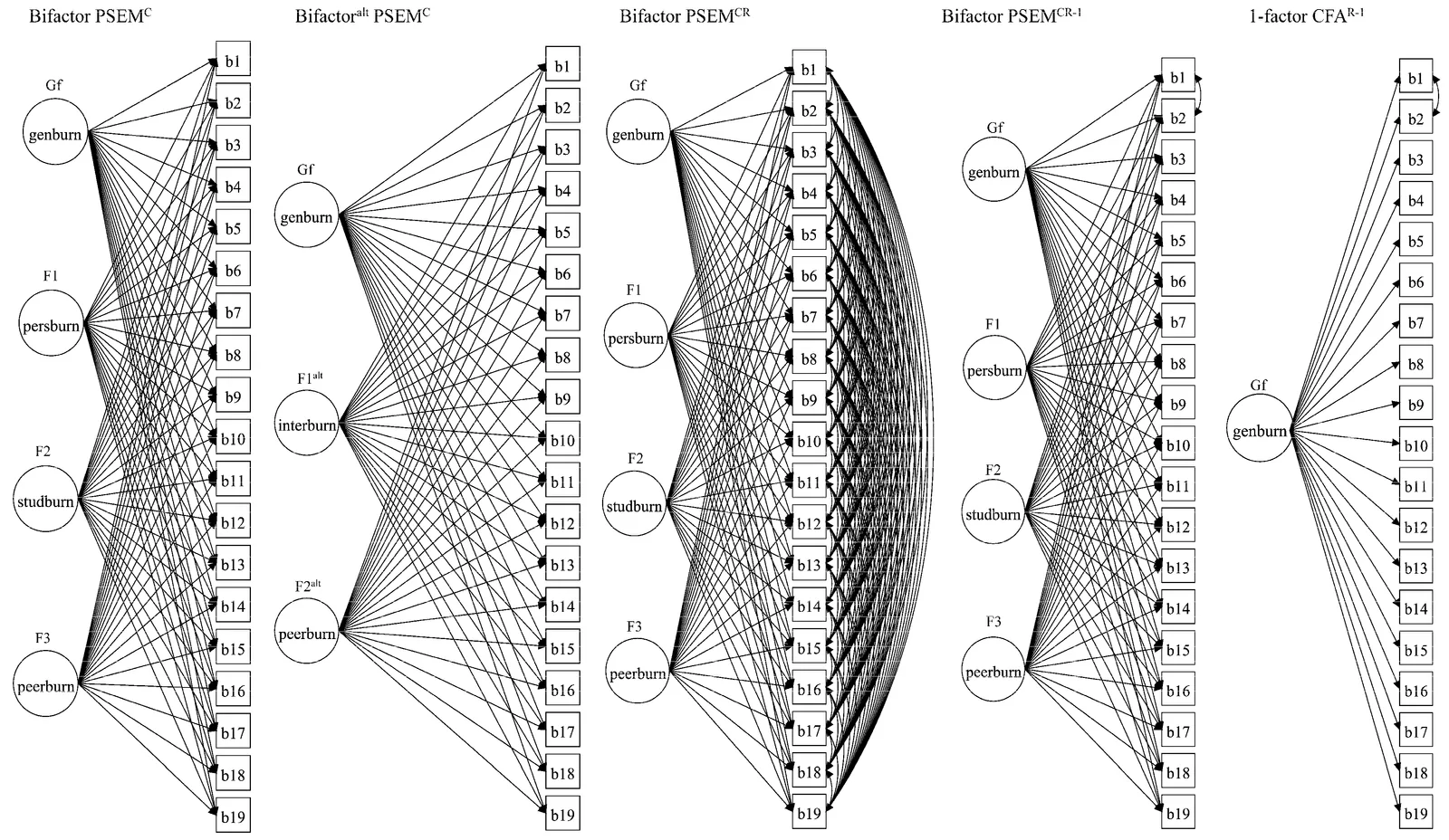
Models Tested, Notes: genburn (Gf), = General burnout factor; persburn (F1) = Personal burnout; studburn (F2) = Study burnout; peerburn (F3) = Peer burnout; interburn (F1^alt^) = persburn and studburn factors combined in the alternative model; peerburn (F2^alt^) = Peer burnout in the alternative model; ^alt^ = alternative bifactor model; ^CR^ = includes item cross-loadings and correlated residuals with ALF (0,1) priors, ^CR−1^ = includes cross-loadings and one correlated residual; ^R−1^ = includes one correlated residual.

**Figure 4:**
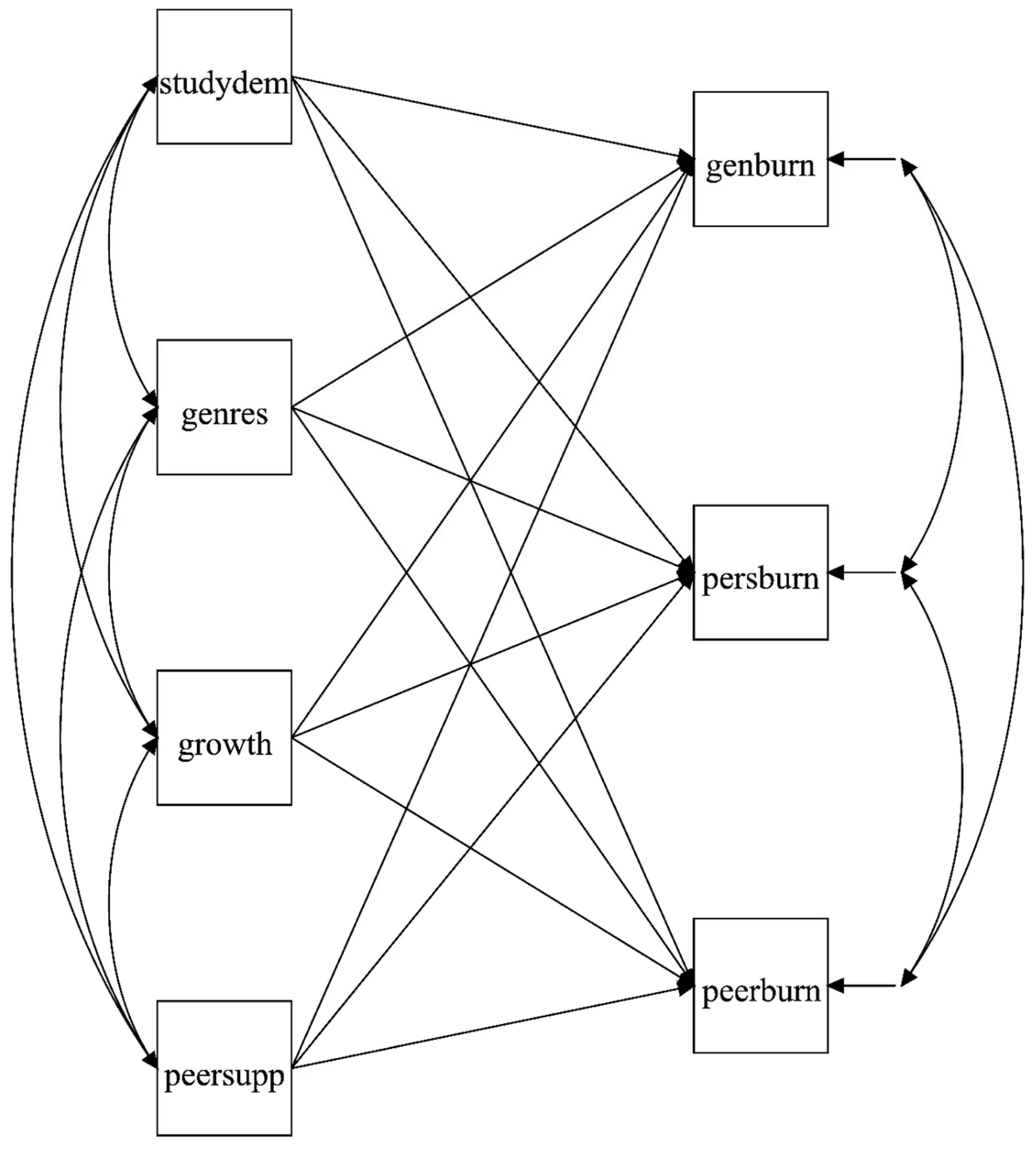
Copenhagen Burnout Inventory regressed on the Study Demands and Study Resources scales. Notes: studydem = Study demands; genres = General resources; peersupp = Peer support; genburn = General burnout factor (Gf); persburn = Personal burnout; peerburn = Peer burnout.

**Figure 5:**
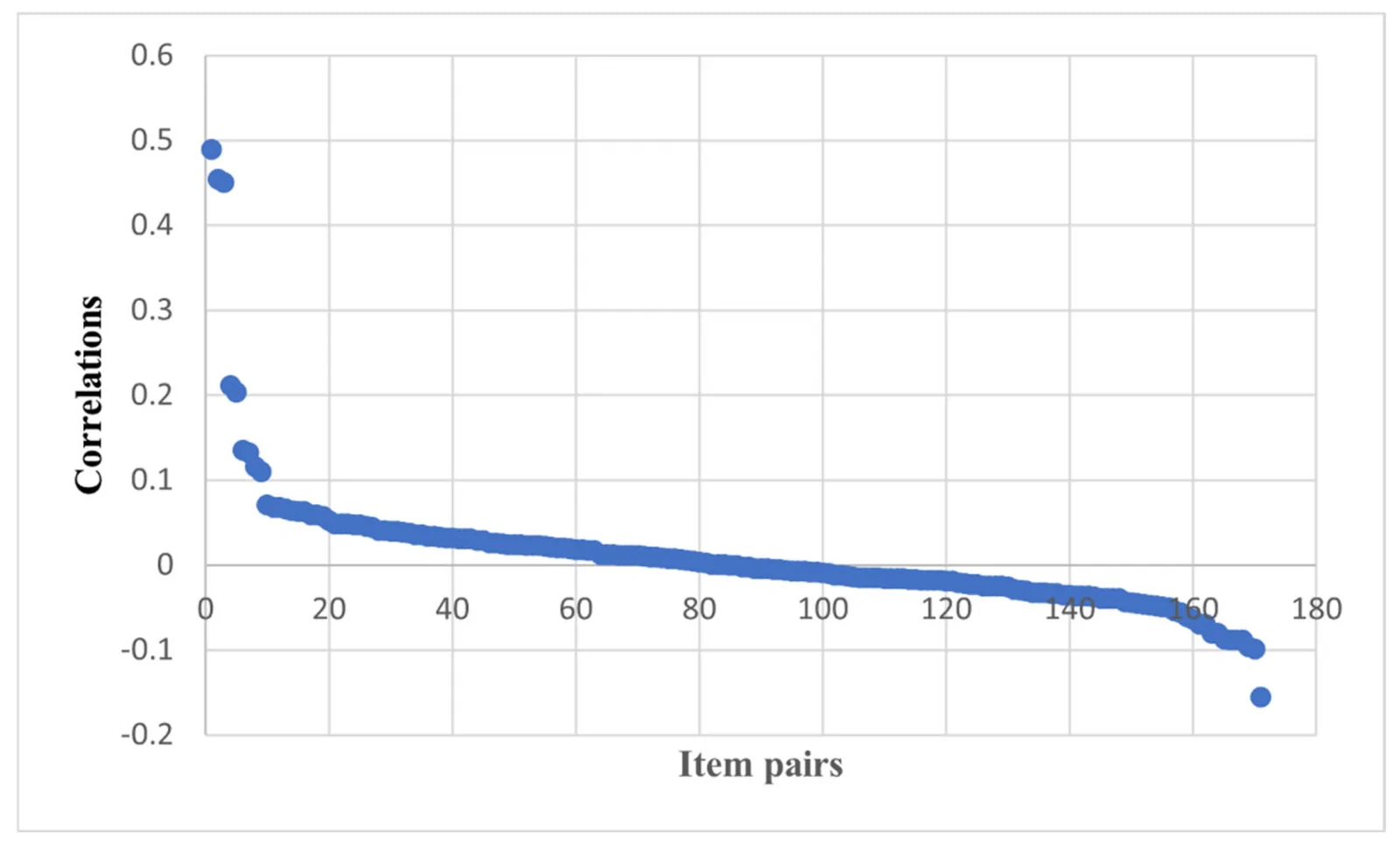
Ranked correlated residuals: Bifactor PSEM^CR^ model.

**Table 1:** CFA and PSEM model fit for the Copenhagen Burnout Inventory.

Model	Chi^2^	D.F.	*p*-Value	RMSEA	CFI	TLI
3-factor CFA	1793.91	149	0.000	0.075 [0.072–0.79]	0.966	0.961
3-factor CFA 2nd Order	1793.91	149	0.000	0.075 [0.072–0.79]	0.966	0.961
2-factor^alt^ CFA	3050.73	171	0.000	0.100 [0.096–0.103]	0.940	0.932
3-factor PSEM^C^	3251.69	117	0.000	0.115 [0.111–0.118]	0.935	0.906
Bifactor PSEM^C^	1631.77	101	0.000	0.086 [0.082–0.089]	0.968	0.947
Bifactor^alt^ PSEM^C^	3250.77	117	0.000	0.116 [0.113–0.119]	0.935	0.906
Bifactor PSEM^CR^	0	0	0.000	0	1.00	1.00
**Bifactor PSEM^CR−1^**	**915.39**	**100**	**0.000**	**0.063 [0.059–0.067]**	**0.983**	**0.971**
1-factor CFA^R−1^	10,851.74	151	0.000	0.191 [0.188–0.194]	0.759	0.727

Notes: CFA = confirmatory factor analysis; d.f. = degrees of freedom; PSEM = penalised structure equation model; CFI = comparative fit index; TLI = Tucker–Lewis index; RMSEA = root mean square error of approximation; ^alt^ = alternative factor model; ^C^ = with cross-loadings; ^CR^ = includes cross-loadings and all correlated residuals with ALF (0,1) priors; ^CR−1^ = includes cross-loadings and one correlated residual; ^R−1^ = includes one correlated residual, [ ] CI = 90% confidence interval; bold font = selected models.

**Table 2:** CFA and PSEM models with cross-loading summary.

	3-Factor CFA Model				2-Factor CFA Model			3-Factor PSEM^C^			
λ Loadings	F1	F2	F3	Res	F1	F2	Res	F1	F2	F3	Res
Primary λ:											
Mean	0.77	0.82	0.75	0.28	0.72	0.81	0.46	0.68	0.78	0.69	0.34
Maximum	0.86	0.87	0.90	0.57	0.85	0.90	0.88	0.86	0.94	0.90	0.57
Minimum	0.62	0.78	0.36	0.13	0.34	0.69	0.29	0.50	0.68	0.05	0.14
Cross λ:											
Mean								0.09	0.07	0.00	
Maximum								0.19	0.29	0.05	
Minimum								−0.15	−0.17	−0.08	
ARPB	0.17	0.14	0.01								
Correlations											
F1	0.90				*0.94*			0.84			
F2	0.78	0.92			0.35	*0.92*		0.69	0.91		
F3	0.31	0.36	0.90					0.23	0.36	0.89	
Sec^Order^ λ:											
Gf	0.82	0.95	0.38								
Res	0.33	0.09	0.86								

Notes: Gf = general factor, F1 = personal burnout, F2 = academic/study burnout, F3 = peer burnout, Primary λ = primary loadings, Cross λ = cross-loadings; ARPB = absolute mean relative % parameter bias, Sec^Order^ = second-order model, Res = residuals; values underlined on the diagonal of the correlation matrix are omega reliabilities for the factors.

**Table 3:** Bifactor loading summary for the Copenhagen Burnout Inventory and auxiliary indices.

	Bifactor PSEM^CR^						Bifactor PSEM^CR−1^				
λ Loadings	Gf	F1	F2	F3	Res	1-Fact	Gf	F1	F2	F3	Res
Primary λ:											
Mean	0.56	0.44	0.01	0.72	0.39	0.65	0.56	0.44	0.02	0.75	0.36
Maximum	0.85	0.56	0.34	0.86	0.88	0.84	0.86	0.54	0.32	0.85	0.88
Minimum	0.25	0.34	−0.37	0.61	0.16	0.30	0.26	0.32	−0.31	0.64	0.13
Cross λ:										/	
Mean		0.01	0.00	0.00				0.01	−0.02	0.00	
Maximum		0.08	0.05	0.04				0.13	0.22	0.05	
Minimum		−0.02	−0.03	−0.05				−0.05	−0.30	−0.05	
Indices:											
ARPB		-	-	-	-	0.54	0.01	0.06	0.11	0.06	
PUC	0.70	-	-	-	-		0.70				
FD	0.97						0.97				
H	0.94						0.94				
ωS	0.95	0.89	0.92	0.90			0.95	0.89	0.93	0.89	
ECV	0.59	0.10	0.04	0.27			0.58	0.10	0.03	0.28	
ECVss		0.35 ^+^	0.10	0.85 ^+^		-		0.36 ^+^	0.09	0.83 ^+^	
ωH/ωHS	0.77	0.31 ^+^	0.00	0.72 ^+^		-	0.77	0.31 ^+^	0.00	0.72 ^+^	
SS_λ_	6.54	1.21	0.47	3.14		-	6.83	1.22	0.39	3.40	

Notes: 1-fact = one-factor CFA model; Gf = general burnout factor; F1 = personal burnout = F2 = study burnout; F3 = peer burnout; Primary λ = primary loadings, Cross λ = cross-loadings; ECV = explained common variance for the proportion of all common variance explained by that factor; ECVss = the proportion of common variance explained by items of the specific factor; ω = omega reliability; ωH/ωHS = omega hierarchical; FD = factor determinacy; H = construct replicability, ARPB = absolute mean relative percentage parameter bias; ^+^ = VAR ≥ 1.1; SS_λ_ = sum of squared item loadings; ^CR^ = includes cross-loadings and all correlated residuals with ALF (0,1) priors; ^CR−1^ = includes cross-loadings and one correlated residual.

**Table 4:** CFA and PSEM model fit for the Students Demands and Resources Scale.

Model	Chi^2^	D.F.	*p*-Value	RMSEA	CFI	TLI
1-factor CFA^R−1^ (D)	47.66	4	0.000	0.075 [0.057–0.095]	0.983	0.959
Bifactor PSEM^c^ (R)	287.75	73	0.000	0.036 [0.031–0.040]	0.991	0.980

Notes: CFA = confirmatory factor analysis; d.f. = degrees of freedom; PSEM = penalised structural equation model; CFI = comparative fit index; TLI = Tucker–Lewis index; RMSEA = root mean square error of approximation; ^R−1^ = includes one correlated residual; ^C^ = includes cross-loadings; [ ] CI = 90% confidence interval; D = Student Demands Scale; R = Student Resources Scale.

**Table 5:** Bifactor loading summary for the Study Demands and Study Resources scales and auxiliary indices.

	Bifactor PSEM^C^						Study Demands	
λ Loadings:	Gf	F1	F2	F3	F4	Res	Gf	Res
Primary λ:								
Mean	0.56	0.38	0.74	0.28	0.21	0.43	0.60	0.62
Maximum	0.84	0.64	0.86	0.45	0.28	0.72	0.75	0.86
Minimum	0.10	0.16	0.56	−0.01	0.11	0.21	0.38	0.44
Cross λ:								
Mean		−0.01	−0.01	0.03	−0.10			
Maximum		0.08	0.07	0.15	0.23			
Minimum		−0.12	−0.04	−0.03	−0.51			
Indices:								
PUC	0.78							
FD	0.95							
H	0.93							
ωS	0.93	0.80	0.84	0.87	0.84		0.75	
ECV	0.65	0.10	0.17	0.06	0.03			
ECVss		0.37 ^+^	0.86 ^+^	0.19 ^+^	0.11			
ωH/ωHS	0.83	0.30 ^+^	0.72 ^+^	0.12 ^+^	0.09			
SS_λ_	6.41	1.00	1.68	0.55	0.18			

Notes; Gf = General resources factor; F1 = Growth; F2 = Peer support; F3 = Lecturer support; F4 = Information accessibility; Primary λ = primary loadings, Cross λ = cross-loadings; ECV = explained common variance for the proportion of all common variance explained by that factor; ECVss = explained common variance for the proportion of common variance explained by items of the specific factor; ω = omega reliability; ωH/ωHS = omega hierarchical; FD = factor determinacy; H = construct replicability; ^+^ = VAR ≥ 1.1; SS_λ_ = sum of squared item loadings; ^C^ = includes cross-loadings.

**Table 6:** Bifactor PSEM models for the Copenhagen Burnout Inventory regressed on the criterion Study Demands and Study Resources scales.

Model	Chi^2^	D.F.	*p*-Value	RMSEA	CFI	TLI
Bifactor PSEM^CR–1^ Criterion	0.00	0	0.000	0.00	1.00	1.00

Notes: PSEM, penalised structural equation model; ^CR−1^ = includes cross-loadings and one correlated residual for the CBI scale.

**Table 7:** Bifactor PSEM^CR−1^ regression coefficients for the Copenhagen Burnout Inventory on the criterion Study Demands and Study Resources scales.

Regression Models	β	S.E.	*p*-Value
Genburn on			
Studydem	0.37	0.03	0.00
Genres	−0.34	0.03	0.00
Growth	−0.10	0.04	0.00
Peersupp	0.02	0.02	0.35
R-square	0.32	0.02	0.00
Persburn on			
Studydem	0.03	0.05	0.59
Genres	0.01	0.03	0.80
Growth	−0.01	0.05	0.97
Peersupp	−0.02	0.03	0.65
R-square	0.00	0.00	0.31
Peerburn on			
Studydem	−0.13	0.03	0.00
Genres	−0.18	0.03	0.00
Growth	0.10	0.03	0.00
Peersupp	−0.50	0.02	0.00
R-square	0.29	0.02	0.00
Residual correlations			
Persburn/genburn	−0.15	0.03	0.00
Peerburn/genburn	−0.03	0.03	0.43
Peerburn/persburn	−0.02	0.03	0.44

Notes: Genburn = General burnout factor; Persburn = Personal burnout; Peerburn = Peer burnout; Studydem = Study demands; Genres = General study resources factor; Growth = Growth resources factor; Peersupp = Peer support factor; ^CR−1^ = includes cross-loadings and one correlated residual.

## Data Availability

The datasets analysed, as well as the Mplus syntax files for selected bifactor PSEMs for the CBI and SDRS and regression analysis with plausible values, are available from the Figshare database accession number(s): https://doi.org/10.6084/m9.figshare.32964434.

## Supplementary Materials

The following supporting information can be downloaded at: https://www.mdpi.com/article/10.3390/bs16101723/s1. File S1: Copenhagen Burnout Instrument; File S2: Study Demands and Study Resources Scale; Table S1: Evidence matrix for EFA/CFA models published in CBI validation studies; Table S2: Trend summary from the evidence matrix of published EFA/CFA models in CBI validation studies; Table S3: Value-added ratio of a factor score (VAR); Table S4: Study Demands and Resources Scale factor models. Reference (Aiello et al., 2022) is cited in the Supplementary Materials.

## Appendix A

**Table A1:** CFA factor models and a PSEM model of the Copenhagen Burnout Inventory with cross-loadings.

	3-Factor CFA				2-Factor^alt^ CFA			3-Factor PSEM^C^			
ITEM	F1	F2	F3	Res	F1^alt^	F2^alt^	Res	F1	F2	F3	Res
B1	**0.76**			0.42	**0.71**		0.49	**0.79**	0.01	−0.02	0.38
B2	**0.76**			0.42	**0.71**		0.49	**0.86**	−0.05	−0.02	0.33
B3	**0.78**			0.40	**0.73**		0.46	**0.68**	0.12	0.02	0.40
B4	**0.85**			0.28	**0.80**		0.36	**0.60**	0.29	−0.01	0.32
B5	**0.86**			0.27	**0.81**		0.35	**0.66**	0.24	0.01	0.30
B6	**0.62**			0.61	**0.58**		0.67	**0.50**	0.12	0.05	0.63
B7		**0.85**		0.29	**0.82**		0.33	0.00	**0.88**	−0.08	0.27
B8		**0.87**		0.24	**0.85**		0.29	−0.06	**0.94**	−0.04	0.21
B9		**0.80**		0.36	**0.77**		0.40	0.00	**0.80**	0.01	0.36
B10		**0.78**		0.39	**0.75**		0.44	0.12	**0.68**	0.00	0.41
B11		**0.81**		0.35	**0.78**		0.40	0.09	**0.72**	0.03	0.37
B12		**0.80**		0.35	**0.77**		0.40	0.12	**0.68**	0.05	0.38
B13		**0.36**		0.87	**0.34**		0.88	*0.15*	**0.20**	0.05	0.88
B14			**0.80**	0.36		**0.80**	0.36	−0.13	0.13	**0.78**	0.36
B15			**0.90**	0.19		**0.90**	0.19	−0.15	0.14	**0.90**	0.17
B16			**0.87**	0.24		**0.87**	0.24	−0.01	0.02	**0.87**	0.24
B17			**0.69**	0.53		**0.69**	0.53	0.02	−0.02	**0.70**	0.52
B18			**0.86**	0.27		**0.86**	0.27	0.19	−0.17	**0.87**	0.24
B19			**0.75**	0.44		**0.75**	0.44	0.14	−0.11	**0.75**	0.44
SE_AVE_	0.01	0.01	0.01	0.01	0.01	0.01	0.02	0.03	0.03	0.02	0.02
SE_MAX_	0.02	0.01	0.02	0.02	0.02	0.01	0.02	0.04	0.04	0.03	0.02
Sec^Order^ λ:											
Gf	**0.82**	**0.95**	**0.38**								
Res	0.33	0.09	0.86								
Correl											
F1	*0.90*							*0.84*			
F2	0.78	*0.92*			0.35			0.68	*0.91*		
F3	0.31	0.36	*0.90*					0.27	0.36	*0.89*	

Notes: Gf = General burnout factor, F1 = Personal Burnout; F2 = Study Burnout; F3 = Peer Burnout; Correl = Correlation Matrix, SEC^Order^ = Second-order model, Res = residuals, Bolded values = Primary factors and statistically significant (*p* ≤ 0.05), Underlined values = substantive cross-loadings and statistically significant (*p* ≤ 0.05), the values on the diagonal of the correlation matrix are omega reliabilities.

**Table A2:** Bifactor PSEM^C^ model for the CBI.

	Bifactor PSEM^C^					Bifactor^alt^ PSEM^C^			
ITEM	Gf	F1	F2	F3	Res	Gf	F1^alt^	F2^alt^	Res
B1	**0.54**	**0.54**	−0.27	−0.01	0.35	**0.57**	**0.55**	−0.01	0.38
B2	**0.52**	**0.60**	−0.24	−0.01	0.31	**0.55**	**0.60**	−0.01	0.33
B3	**0.62**	**0.49**	−0.02	0.03	0.39	**0.62**	**0.47**	0.02	0.40
B4	**0.72**	**0.41**	0.02	0.00	0.31	**0.72**	**0.41**	−0.01	0.32
B5	**0.71**	**0.46**	0.03	0.01	0.28	**0.71**	**0.45**	0.01	0.30
B6	**0.50**	**0.36**	0.00	0.05	0.62	**0.50**	**0.34**	0.05	0.63
B7	**0.86**	0.01	**0.12**	−0.07	0.24	**0.85**	**−0.04** ^#^	−0.08	0.27
B8	**0.90**	−0.05	**0.12**	−0.04	0.18	**0.89**	**−0.09** ^#^	−0.04	0.21
B9	**0.81**	−0.03	**−0.04** ^#^	0.00	0.35	**0.80**	**−0.04** ^#^	0.00	0.36
B10	**0.75**	0.03	**−0.23**	−0.01	0.39	**0.77**	**0.06** ^#^	0.00	0.41
B11	**0.74**	−0.01	**−0.45**	0.01	0.25	**0.80**	**0.04** ^#^	0.02	0.37
B12	**0.73**	0.02	**−0.43**	0.04	0.29	**0.78**	**0.06** ^#^	0.04	0.38
B13	**0.33**	0.11	**0.01** ^#^	0.04	0.88	**0.32**	**0.10**	0.04	0.88
B14	**0.33**	0.01	0.20	**0.72**	0.33	**0.33**	−0.09	**0.72**	0.36
B15	**0.36**	0.00	0.20	**0.83**	0.13	**0.36**	−0.11	**0.83**	0.17
B16	**0.33**	0.01	0.00	**0.81**	0.24	**0.33**	0.00	**0.81**	0.24
B17	**0.25**	−0.03	−0.10	**0.64**	0.51	**0.25**	0.02	**0.65**	0.52
B18	**0.28**	0.02	−0.29	**0.81**	0.18	**0.28**	0.15	**0.81**	0.24
B19	**0.26**	−0.01	−0.27	**0.68**	0.40	**0.26**	0.11	**0.69**	0.44
SE_AVE_	0.02	0.02	0.02	0.02	0.02	0.03	0.05	0.01	0.02
SE_MAX_	0.03	0.03	0.03	0.02	0.02	0.06	0.08	0.02	0.02

Gf = General factor, F1 = Personal Burnout; F2 = Study burnout; F3 = Peer Burnout; F1^alt^ (interburn) F1 and F2 factor combined; F2^alt^ = Peer Burnout for the alternative model; SE_AVE_ = Mean standard errors for item loading estimations; SE_MAX_ = Maximum standard error for item loading estimations; Bolded values = Primary factors and statistically significant (*p* ≤ 0.05), Underlined values = substantive cross-loadings and statistically significant (*p* ≤ 0.05), ^#^ = Not statistically significant (*p* > 0.05); ^C^ = all cross-loadings included with ALF (0,1) priors; Res = Residuals.

**Table A3:** Bifactor PSEM models for the Copenhagen Burnout Inventory.

	Bifactor PSEM^CR^					1-Fact	Bifactor PSEM^CR−1^				
ITEM	Gf	F1	F2	F3	Res		Gf	F1	F2	F3	Res
B1	**0.59**	**0.34**	−0.03	−0.01	0.54	**0.36**	**0.59**	**0.32**	−0.07	−0.01	0.55
B2	**0.56**	**0.43**	−0.02	−0.01	0.50	**0.40**	**0.56**	**0.40**	−0.05	−0.01	0.52
B3	**0.61**	**0.56**	0.01	0.03	0.32	**0.31**	**0.60**	**0.54**	−0.01	0.03	0.35
B4	**0.70**	**0.44**	0.01	0.00	0.32	**0.30**	**0.69**	**0.48**	0.02	0.00	0.29
B5	**0.69**	**0.49**	0.01	0.01	0.28	**0.70**	**0.69**	**0.53**	0.03	0.02	0.24
B6	**0.49**	**0.41**	0.01	0.04	0.59	**0.70**	**0.49**	**0.40**	0.01	0.05	0.60
B7	**0.82**	0.02	**0.31**	−0.05	0.22	**0.72**	**0.82**	0.05	**0.30**	−0.05	0.24
B8	**0.85**	−0.01	**0.34**	−0.01	0.16	**0.79**	**0.86**	−0.02	**0.32**	−0.01	0.16
B9	**0.80**	−0.01	**0.12**	0.00	0.35	**0.80**	**0.80**	−0.01	**0.13**	0.01	0.35
B10	**0.78**	0.01	**−0.10**	−0.01	0.38	**0.57**	**0.78**	0.02	**−0.08**	−0.02	0.38
B11	**0.81**	−0.02	**−0.37**	−0.01	0.21	**0.82**	**0.82**	−0.05	**−0.31**	−0.02	0.24
B12	**0.80**	0.00	**−0.31**	0.02	0.27	**0.84**	**0.80**	−0.01	**−0.29**	0.02	0.28
B13	**0.33**	0.08	**0.05** ^#^	0.03	0.88	**0.77**	**0.32**	0.13	**0.05** ^#^	0.05	0.88
B14	**0.30**	0.01	0.02	**0.67**	0.46	**0.74**	**0.29**	0.02	0.21	**0.74**	0.33
B15	**0.33**	0.00	0.01	**0.78**	0.28	**0.76**	**0.32**	0.00	0.22	**0.85**	0.13
B16	**0.33**	0.01	−0.01	**0.86**	0.16	**0.76**	**0.32**	0.00	0.00	**0.81**	0.24
B17	**0.25**	−0.01	0.01	**0.64**	0.52	**0.34**	**0.26**	−0.02	−0.10	**0.64**	0.51
B18	**0.32**	0.02	−0.02	**0.75**	0.34	**0.55**	**0.32**	0.03	−0.30	**0.79**	0.17
B19	**0.29**	0.01	−0.03	**0.61**	0.54	**0.56**	**0.30**	0.01	−0.29	**0.66**	0.39
SE_AVE_	0.02	0.01	0.01	0.01	0.02	0.01	0.02	0.02	0.02	0.02	0.02
SE_MAX_	0.03	0.03	0.03	0.02	0.02	0.02	0.03	0.03	0.03	0.02	0.02
Correl^R^:											
B1/B2		0.49				0.54		0.50			
B14/B15				0.45							
B18/B19				0.45							
B17/B18				0.21							

Gf = general factor, F1 = personal burnout; F2 = study burnout; F3 = peer burnout; SE_AVE_ = mean standard errors for item loading estimations; SEmax = maximum standard error for item loading estimations; Correl^R^ = correlated residuals coefficients for item pairs; Bolded values = Primary factors and statistically significant (*p* ≤ 0.05), Underlined values = substantive cross-loadings and statistically significant (*p* ≤ 0.05), ^#^ = Not statistically significant (*p* > 0.05); ^CR^ = all cross-loadings and correlated residual included with ALF (0,1) priors; ^CR−1^ = all cross-loadings and one correlated residual freed.
