# Supplementary material for: The Dimensionality of the 19-Item Copenhagen Burnout Inventory Adapted for Students: A Bifactor Penalised SEM Analysis
Source: Behav Sci (Basel). 2026 Sep 22;16(10):1723. doi: 10.3390/bs16101723 (PMC13701781; doi:10.3390/bs16101723)
Supplement: Supplementary file 1 [file behavsci-16-01723-s001.zip › behavsci-4473320-supplementary.pdf]

Supplementary Materials (Click on item)

Contents

Table S1: Evidence matrix for EFA/CFA models published in CBI validation studies (Use the PDF zoom function to view)........ 2

Table S2: Trend summary from the evidence matrix of published EFA/CFA models in CBI validation studies 4

File S1: Copenhagen Burnout Instrument........ 5

File S2: Study Demands and Study Resources Scale 7

Table S3: Value-added ratio of a factor score (VAR)........ 8

Table S4: Study Demands and Resources Scale factor models 9

Table S1: Evidence matrix for EFA/CFA models published in CBI validation studies (Use the PDF zoom function to view)

No.	Article	CBI version/scale	Design/sample	Accepted factor model(s)	Model fit indices	Factor loadings range and mean	Factor intercorrelations	Freed correlated residuals & removed items	Scale reliabilities	External variables used for criterion/convergent validity	Notes / interpretation
1	Kristensen, Borritz, Villadsen & Christensen (2005) — The Copenhagen Burnout Inventory: A new tool for the assessment of burnout. Work & Stress, 19(3), 192–207. DOI: 10.1080/02678370500297720	Original CBI, 19-item occupational version; three scales: personal burnout (PB), work-related burnout (WB), client-related burnout (CB).	Instrument-development and validation study with prospective follow-up; PUMA human-service employees, baseline N=1,914.	No CFA/SEM factor model reported; three theoretically defined CBI scales retained: personal burnout (PB), work-related burnout (WB), client-related burnout (CB).	Not reported / not applicable.	Not reported / not applicable.	PB–WB=.72; PB–CB=.46; WB–CB=.61.	No freed residuals; no removed items. One inverse-scored WB energy item retained.	Cronbach α: PB=.87; WB=.87; CB=.85.	SF-36 vitality, mental health, general health; job satisfaction; choosing same job again; prospective sickness absence, sickness spells, sleep problems, weekly painkiller use, intention to quit.	Foundational study; strong reliability and clear criterion/predictive validity but no formal CFA fit indices.
2	Campos, Carlotto & Marôco (2013) — Copenhagen Burnout Inventory–Student Version: Adaptation and Transcultural Validation for Portugal and Brazil. Psicologia: Reflexão e Crítica, 26(1), 87–97. DOI: 10.1590/S0102-79722013000100010	CBI-S / CBI-Student Version for college students; four scales: PB, studies-related burnout (SRB), colleagues-related burnout (CRB), teachers-related burnout (TRB); refined version after removing two items.	Cross-sectional adaptation, transcultural validation and multigroup psychometric study; Brazilian (n=958) and Portuguese (n=556) higher education students.	Four-factor CBI-S accepted after refinement: PB, studies-related burnout (SRB), colleagues-related burnout (CRB), teachers-related burnout (TRB). Cross-cultural invariance between Brazil and Portugal not supported.	Initial: χ^2/df=5.473, CFI=.928, GFI=.889, RMSEA=.067. Refined: χ^2/df=6.094, CFI=.931, GFI=.889, RMSEA=.071. Test/validation: χ^2/df=4.602, CFI=.928, GFI=.876, RMSEA=.049. Brazil/Portugal multigroup: χ^2/df=5.459, CFI=.907, GFI=.858, RMSEA=.054.	After refinement all λ≥.50; mean not reported.	Latent factor correlations reported for Global Sample as PB–SRB=.87; PB–CRB=.35; PB–TRB=.41; SRB–CRB=.35; SRB–TRB=.55; CRB–TRB=.45.. Observed subscale correlations: PB–SRB=.78; PB–CRB=.32; PB–TRB=.37; SRB–CRB=.30; SRB–TRB=.50; CRB–TRB=.41.	Removed item 10 (SRB; reversed item) and item 22 (TRB) due to λ<.50/negative loading. No freed residual pairs reported.	Cronbach α: PB=.901; SRB=.875; CRB=.910; TRB=.931; total=.957. Composite reliability: PB=.903; SRB=.877; CRB=.913; TRB=.931.	MBI-SS emotional exhaustion, cynicism, professional efficacy; Beck Depression Inventory (BDI).	Good student-version reliability and criterion associations; item removal and lack of Brazil/Portugal metric stability indicate adaptation sensitivity. PB-SRB overlap is high
3	Marôco & Campos (2012) — Defining the Student Burnout construct: A structural analysis from three Burnout Inventories. Psychological Reports: Human Resources & Marketing, 111(3), 814–830. DOI: 10.2466/14.10.20.PR0.111.6.814-830	CBI-SS / student version included as one of three burnout inventories; four CBI-SS scales: PB, SRB, CRB and TRB.	Cross-sectional SEM construct-definition study with college students; CBI-SS analysed alongside MBI-SS and OLBI-SS.	Second-order student burnout model accepted. Burnout was represented by lower-order dimensions from MBI-SS, CBI-SS and OLBI-SS. CBI-SS maintained four factors.	Overall measurement model: χ^2/df=2.7, CFI=.94, GFI=.89, RMSEA=.04 (90% CI=.040–.043). CBI-SS submodel: χ^2/df=5.95, CFI=.93, GFI=.89, RMSEA=.07 (90% CI=.067–.074).	CBI-SS standardized loadings approximately .63–.94; mean approximately .80 from figure. Subscale ranges: PB≈.64–.86; SRB≈.73–.82; CRB≈.63–.85; TRB≈.75–.94.	CBI-SS r^2 values: PB–SRB=.57; PB–CRB=.12; PB–TRB=.16; SRB–CRB=.10; SRB–TRB=.26; CRB–TRB=.18. Second-order paths from burnout: CBI-SRB=.97; CBI-PB=.86; CBI-TRB=.55; CBI-CRB=.39.	Correlated item errors were allowed for errors caused by the same factor, PB_C1-PB_C2, SB_C8-SBC9, CB_C14-CBC15, CB_C18-CB_C19, TB_C20-TB_C21. Removed CBI-SS item 12; also removed MBI items 3 and 14.	CBI-SS: PB α=.90, CR=.90, AVE=.61; SRB α=.88, CR=.88, AVE=.55; CRB α=.91, CR=.91, AVE=.63; TRB α=.93, CR=.93, AVE=.73.	Other burnout inventories: MBI-SS and OLBI-SS used as convergent structural comparators.	Supports exhaustion-related dimensions as the core of student burnout, especially personal and study-related burnout. PB-SRB overlap high
4	Seisembekov et al. (2025) — Validation of the Russian version of the Copenhagen Burnout Inventory among nurses in Kazakhstan and Kyrgyzstan. International Journal of Nursing Sciences, 12, 176–183. DOI: 10.1016/j.ijnss.2025.02.004	Russian CBI (R-CBI), 19-item occupational version; PB, WB and CB.	Cross-sectional translation and validation study; nurses in Kazakhstan and Kyrgyzstan, total N=1,530.	Three-factor R-CBI accepted after model modification; 19 items retained.	Initial CFA: Kazakhstan CFI=.884, TLI=.867, RMSEA=.091; Kyrgyzstan CFI=.891, TLI=.875, RMSEA=.085. Modified: Kazakhstan CFI=.922, TLI=.906, RMSEA=.077; Kyrgyzstan CFI=.914, TLI=.899, RMSEA=.077.	Exact loading range/mean not reported in extracted text; CFA supported three-factor structure.	Kazakhstan PB–WB=.89; PB–CB=.52; WB–CB=.75. Kazakhstan PB–WB=.86; PB–CB=.49; WB–CB=.77.	Residual covariances added among selected PB, WB and CB item errors (Kazakhstan: PB1-PB2, PB2-PB3, WRB2-WRB3, CRB1-CRB4, CRB1-CRB5, CRB4-CRB5. Kyrgyzstan: PB1-PB2, PB4-PB5, CRB4-CRB5)	Total Cronbach α=.926 in Kazakhstan and .922 in Kyrgyzstan; subscale α range=.830–.898.	Job satisfaction questionnaire; DASS depression, anxiety and stress; WHO-5 Well-Being Index; demographic/occupational predictors including COVID-19 infection history.	Validation supports reliability and expected distress/well-being associations; fit improved with residual covariances. PB-WB overlap high
5	Walters, Brown & Jones (2018) — Use of the Copenhagen Burnout Inventory with Social Workers: A Confirmatory Factor Analysis. Human Service Organizations: Management, Leadership & Governance, 42(5), 437–456. DOI: 10.1080/23303131.2018.1532371	Original CBI, 19-item occupational version; PB, WB and CB.	Cross-sectional CFA validation study; U.S. social workers, N=1,774, including direct-service, supervisory and other roles.	Original correlated three-factor model adequate; post-hoc three-factor model with four residual covariances accepted. Higher-order model had equivalent fit but authors cautioned against a total-score interpretation.	Model 1: χ^2(149)=1248.04, CFI=.908, TLI=.894, RMSEA=.093. Two-factor: χ^2(151)=1406.15, CFI=.895, TLI=.881, RMSEA=.098. Post-hoc model: χ^2(145)=674.015, CFI=.956, TLI=.948, RMSEA=.065. Cross-validation post-hoc: CFI=.952, TLI=.943, RMSEA=.068.	Standardized loadings=.601–.853; mean≈.775. PB≈.644–.853; WB≈.601–.848; CB≈.674–.836.	Observed: PB–WB=.87; PB–CB=.50; WB–CB=.61. Latent PB–WB was very high (≈.943 in original model; ≈.96 in post-hoc figure).	Freed residuals: PB1–PB2, WB5–WB6, CB1–CB2, CB5–CB6. Removed items: none.	Cronbach α: PB=.902; WB=.906; CB=.897.	No external criterion scale. Invariance/group comparisons by supervisor/non-supervisor, direct-service/non-direct-service and four role categories.	CBI works well for social workers; PB–WB overlap is very high, supporting subscale-level rather than total-score interpretations.
6	Krishnappa et al. (2024) — Evaluation of the psychometric properties of the Copenhagen burnout inventory–student survey (CBI-SS) among health profession educational students at a university in South India. Journal of Medical Education Development, 17(56), 117–128. DOI: 10.61186/edcj.17.56.117	CBI-SS, 25-item student survey; four domains: PB, studies-related burnout, colleagues-related burnout and teachers-related burnout.	Cross-sectional psychometric validation study; undergraduate and postgraduate health-profession students in South India, n=523.	Four-dimensional CBI-SS accepted; all 25 items retained, though EFA indicated some item redistribution between personal and study-related factors.	KMO>.90; Bartlett χ^2=8880.727, df=300, p<.0001. CFA: TLI=.919; RMSEA=.068. Other fit indices named but not all values reported in extracted text.	Exact loading range/mean not reported; all items retained based on EFA/CFA.	Exact coefficients not reported; authors describe moderate-to-good correlations among four subscales.	No freed residuals reported. No items removed. Item 10 retained as reverse-scored.	Cronbach α: subscales approximately .778–.895; total α=.936. Appendix after-FA values: total CBI-SS α = .937; personal, study-related and colleague-related α = .933; teacher-related α = .932.	Known-groups/descriptive comparisons: undergraduate vs postgraduate status; dental vs allied-health PG students; burnout severity categories.	Initial Indian CBI-SS evidence is supportive, but factor details and external validity testing are limited.
7	AL-Qadri et al. (2026) — Assessing the psychometric properties of the Copenhagen Burnout Inventory (CBI) across various sectors in Sudan. Journal of Psychology in Africa, 36(1), 65–77. DOI: 10.32604/jpa.2025.069675	Arabic CBI occupational version; sector-specific 19-item and 17-item forms; PB, WB and CB.	Cross-sectional multi-sector psychometric validation study; Sudanese teachers, healthcare workers and bank employees, N=1,068.	Three-factor Arabic CBI supported. Final forms differed by sector: 19-item version for teachers and bank employees; 17-item version for healthcare workers.	Modified fit: Teachers χ^2/df=2.854, CFI=.936, GFI=.921, TLI=.925, RMSEA=.065. Healthcare χ^2/df=2.942, CFI=.944, GFI=.898, TLI=.934, RMSEA=.077. Bank χ^2/df=3.089, CFI=.952, GFI=.891, TLI=.944, RMSEA=.080. Overall χ^2/df=4.811, CFI=.967, GFI=.922, TLI=.961, RMSEA=.060.	EFA loading ranges: teachers PB=.535–.984, WB=.751–.995, CB=.940–.952; healthcare PB=.762–.899, WB=.690–.862, CB=.444–.819; bank PB=.419–.778, WB=.401–.718, CB=.503–.812. Means not reported.	CBI subscales: PB–WB=.729; PB–CB=.365; WB–CB=.655.	Teachers freed residuals: items 1–2, 8–9, 9–11, 16–18, 15–19. Healthcare: removed WB items 9 and 13; freed residuals 15–16 and 18–19. Bank: freed residuals 1–2, 2–6, 12–13. Overall: freed residuals 1–2 and 15–19.	Reliability was satisfactory across sectors. Overall: ω PB=.925, WB=.830, CB=.942; α PB=.924, WB=.810, CB=.945; CR PB=.804, WB=.738, CB=.813; AVE PB=.909, WB=.882, CB=.914.	Maslach Burnout Inventory domains: emotional exhaustion (EE), personal realization/accomplishment (PR), depersonalization (D), and IPS as reported.	Cross-sector results support CBI use, but item functioning differs by sector, especially healthcare workers.
8	Amaya-Arias (2026) — Assessing burnout in Colombian healthcare professionals: Psychometric properties of the Copenhagen Burnout Inventory (CBI). Journal of Health Psychology, 31(6), 2380–2398. DOI: 10.1177/13591053251387819	Colombian/Spanish CBI occupational version; 19-item original compared with 18-item refined version; PB, WB and CB.	Cross-sectional psychometric validation and IRT study; Colombian healthcare workers, n=510.	Original 3-factor and second-order hierarchical models evaluated. Best practical solution: 18-item model excluding item 13 due low loading/high error.	19-item original: CFI=1.000, TLI=1.003, NFI=.992, RMSEA=.049, χ^2=333.7, df=149. 18-item original: CFI=.994, TLI=.993, NFI=.992, RMSEA=.055, χ^2=335.3, df=132. 18-item hierarchical: CFI=.995, TLI=.994, NFI=.993, RMSEA=.054, χ^2=364.4, df=147. The article has a minor internal inconsistency: the abstract states RMSEA≤ .05 after removing item 13,	Most loadings high but exact range not fully reported in extracted text. Item 13 loading≈.23–.24; second-order paths: PB=.69, WB=.78, CB=.70.	Original model: PB–WB=.90; PB–CB=.66; WB–CB≈.81. Hierarchical residual correlations: PB–WB=.81; PB–CB=.34; WB–CB=.59.	Removed/recommended removal: item 13, "Do you have enough energy for family and friends during leisure time?". No freed correlated residuals reported.	Omega: PB=.92; WB=.89; CB=.90. Cronbach α: PB=.897; WB=.850; CB=.860. Removing item 13 improved WB alpha from .85 to .88.	No independent external criterion variables; IRT and subscale correlations used as psychometric evidence.	Strong support for CBI in Colombian healthcare workers, but item 13 is clearly problematic.
11	Živanović et al. (2021) — Psychometric Properties and Structural Validity of the Serbian Version of the Copenhagen Burnout Inventory. SAGE Open, 11(4), 1–12. DOI: 10.1177/21582440211048889	Serbian CBI short form; final 13-item occupational version; PB, WB and CB, with item 13 placement tested.	Cross-sectional CFA validation and short-form development study; Serbian workers.	Three-factor 13-item short form accepted. Four-factor model slightly better but rejected because latent factors were extremely correlated. Item 13 tested as WB or PB; rephrasing suggested.	Final three-factor I: χ^2(62)=195.12, CFI=.948, TLI=.934, RMSEA=.075, SRMR=.054. Final three-factor II: χ^2(62)=184.81, CFI=.952, TLI=.939, RMSEA=.072, SRMR=.050. Four-factor: χ^2(59)=174.57, CFI=.954, TLI=.940, RMSEA=.072, SRMR=.049.	Three-factor I loadings=.498–.841; mean≈.728. Three-factor II loadings=.521–.839; mean≈.726.	Three-factor I: PB–WB=.937; PB–CB=.617; WB–CB=.781. Three-factor II: PB–WB=.935; PB–CB=.613; WB–CB=.800.	Removed items: 1, 2, 8, 11, 15, 18. Freed residuals in preliminary domain models: 1–2, 7–8, 8–9, 11–12, 14–15, 18–19. Item 13 remained conceptually problematic.	Full 19-item α: PB=.893, WB=.887, CB=.888, total=.941. Final 13-item α: PB≈.848–.861, WB≈.812–.819, CB=.824, total=.912.	No external criterion variables.	Shows that the original 19-item CBI can contain locally dependent/redundant items; short forms can improve parsimony.
12	Tran et al. (2023) — Psychometric properties and factor structure of the Vietnamese Copenhagen Burnout Inventory. Journal of Occupational Health, 65(1), e12425. DOI: 10.1002/1348-9585.12425	Vietnamese CBI, 19-item occupational version; PB, WB and CB.	Cross-sectional psychometric validation with test–retest component; hospital nurses in Vietnam, n=587.	Original 19-item three-factor model retained with correlated residuals. 18-item models did not improve fit; W7 flagged but retained.	19-item original: NNFI=.87, TLI=.87, CFI=.89, SRMR=.05, RMSEA=.09, CMIN/df=6.35. 19-item with covariances: NNFI=.91, TLI=.91, CFI=.93, SRMR=.05, RMSEA=.08, CMIN/df=4.74.	All CFA loadings exceeded .60 in modified model; mean not reported. W7 showed low/atypical loading in EFA and formed a single-item fourth factor.	PB–WB=.63; PB–CB=.53; WB–CB=.68.	Freed residuals: P1–P2, W1–W2, C5–C6. Removed items: none.	Cronbach α: PB=.91; WB=.87; CB=.88. Test–retest ICCs over 3 months were low: PB≈.16, WB≈.24, CB≈.29.	DASS depression, anxiety and stress; HR-QOL; job performance; Utrecht Work Engagement Scale (UWES).	Convergent validity was strongest for distress and work engagement; local residuals improved fit while preserving 19-item CBI.
13	Youssef, Abou-Abbas & Youssef (2022) — Feeling the burn in the era of COVID-19: Cross-cultural adaptation and validation of the Arabic version of the Copenhagen Burnout Inventory among community pharmacists. Journal of Pharmaceutical Policy and Practice, 15, Article 21. DOI: 10.1186/s40545-022-00419-x	Arabic version of the original CBI, 19-item occupational version; PB, WB and CB.	Cross-sectional cross-cultural adaptation and validation study during COVID-19; Lebanese community pharmacists.	Three-factor Arabic CBI accepted after one residual covariance between two personal-burnout items.	One-factor poor: χ^2(195)=2013, CMIN/df=10.32, RMSEA=.111, SRMR=.089, CFI=.683. Three-factor default: χ^2(121)=843.34, CMIN/df=7.147, RMSEA≈.072, SRMR=.055, CFI=.902. Modified three-factor: χ^2(117)=522.37, CMIN/df=4.46, RMSEA=.041, SRMR=.043, GFI=.918, CFI=.917, NFI=.927, TLI=.911.	EFA salient loadings: PB≈.691–.782; WB≈.707–.881; CB≈.618–.801; overall≈.618–.881. Mean not reported.	PB–WB=.212; PB–CB=.297; WB–CB=.377.	Freed residual: PB5–PB6 ("worn out" with "weak/susceptible to illness"). Removed items: none.	Cronbach α: total≈.868; PB=.842; WB=.902; CB=.774.	Hospital Anxiety and Depression Scale (HADS); demographic/work variables.	Low subscale correlations were unexpected; the appropriateness of using principal component analysis with varimax rotation resulted in low subscale correlations is questionable; HADS anxiety/depression relations were substantial, particularly for personal burnout.
15	Moser et al. (2023) — Psychometric properties of the Brazilian Portuguese version of the Copenhagen Burnout Inventory (CBI) in healthcare professionals. Trends in Psychiatry and Psychotherapy, 45, e20210362. DOI: 10.47626/2237-6089-2021-0362	Brazilian Portuguese CBI, 19-item occupational version; PB, WB and CB; EFA suggested PB+WB combined plus CB.	Cross-sectional reliability and exploratory factor-structure study; Brazilian healthcare professionals during COVID-19.	Exploratory factor analysis suggested two factors: PB+WB combined and CB separate.	KMO=.95; Bartlett p<.001. No CFA fit indices reported.	Factor loadings not reported in extract; supplementary material referenced. Mean not reported.	Factor correlations not reported. Two-factor result suggests strong PB/WB overlap.	No freed residuals; no removed items reported.	Reliability: PB α=.9215; WB α=.8910; CB α=.9296; PB+WB α=.9457. Split-half Spearman-Brown reliability: PB=.9125; WB=.8761; CB=.912; PB+WB=.9382. The spreadsheet appears to use the specific table values correctly, but the article itself is internally inconsistent:	No independent external criterion variables; study focused on reliability and EFA structure.	Very high reliability; PB and WB again highly overlapped in healthcare professionals.
16	Rocha et al. (2020) — Burnout syndrome in university professors and academic staff members: Psychometric properties of the Copenhagen Burnout Inventory–Brazilian version. Psicologia: Reflexão e Crítica, 33(1), Article 11. DOI: 10.1186/s41155-020-00151-y	CBI-Br, Brazilian Portuguese CBI; refined 18-item occupational version after item 10 removal; PB, WB and CB.	Cross-sectional psychometric validation with test/validation split and demographic/occupational associations; Brazilian public university professors and academic staff, n=676.	Refined 18-item three-factor model accepted; second-order hierarchical model also acceptable.	Original 19-item: χ^2/df=7.87, CFI=.92, TLI=.90, RMSEA=.10. Refined 18-item: χ^2/df=6.09, CFI=.95, TLI=.94, RMSEA=.09. SOHM: χ^2/df=6.12, CFI=.95, TLI=.94, RMSEA=.09. Test/validation simultaneous: χ^2/df=3.59, CFI=.95, TLI=.94, RMSEA=.06.	Original λ=.20–.94. Refined λ=.71–.94; mean not reported.	Refined: PB–CB=.50; WB–CB=.59; PB–WB=.92. Second-order paths: PB β=.91, WB β=1.00; CB β=0.58	Removed item 10 (only inverted item; λ=.202). Freed residuals: e1–e2 (LM=128.36), e8–e9 (LM=91.61), e18–e19 (LM=87.11).	Refined: CR PB=.94, WB=.91, CB=.95; α PB=.94, WB=.91, CB=.95. AVE PB=.73, WB=.64, CB=.74.	Gender, work function, age, hours worked per week and duration of employment.	Strong university-staff validation; item 10 and PB–WB overlap are notable.
17	Cerela-Boltunova, Millere & Trups-Kalne (2025) — Adaptation of the Copenhagen Burnout Inventory in Latvia: Psychometric Data and Factor Analysis. International Journal of Environmental Research and Public Health, 22(5), 761. DOI: 10.3390/ijerph22050761	Latvian adaptation of the original CBI, 19-item occupational version; PRB, WRB and CRB.	Cross-sectional adaptation and psychometric factor-analysis study; healthcare workers in Latvia, n=288.	Three-factor model evaluated with EFA/CFA. Overall evidence supported Latvian CBI, but item 10 had a negative loading/correlation and requires attention.	CFA/goodness fit: χ^2/df=2.60; CFI=.997; TLI=.997; NNFI=.997; NFI=.994; RFI=.994; IFI=.997; PNFI=.866. SRMR=.048; RMSEA=.064 (90% CI .055–.074). Robust RMSEA=.139, indicating poor fit. However, abstract reports lower rounded values, CFI = .95 and TLI = .94. The table should add a note that the article contains two inconsistent CFI/TLI presentations.	CFA β: item 10=-.572; all other items β=.621–.956; mean positive β≈.838. EFA variance explained≈68%; KMO=.942.	PRB–WRB=.81; PRB–CRB=.50; WRB–CRB=.92.	No freed residual pairs reported. No items removed in final model, but item 10 flagged: negative item-total correlation=-.489, α would increase to .914 if removed; item 17 also lowe but less problematic.	Overall McDonald's ω=.948; overall α=.938. Subscale α: PRB=.920, WRB=.791, CRB=.884. CFA CR/AVE: PRB=.91/.64; WRB=.88/.60; CRB=.87/.58.	No external psychological criterion scales; demographic/workload descriptives included.	Excellent model fit except robust RMSEA; strong reliability; item 10 again behaves anomalously.
19	Lapa, Carvalho, Viana, Ferreira, Pinto-Gouveia & Cabete (2018) — Development and Evaluation of a Global Burnout Index Derived from the Use of the Copenhagen Burnout Inventory in Portuguese Physicians. Acta Médica Portuguesa, 31(10), 534–541. DOI: 10.20344/amp.10407	Portuguese CBI for physicians; original three CBI domains: personal, work-related and patient-related burnout; also tested a second-order/global burnout index.	Cross-sectional CFA and criterion-validity study; Portuguese physicians, N=1,348.	Three-factor CBI improved by correlated residuals and a second-order/global burnout model accepted; second-order model showed the best fit.	Model 1 three-factor: χ^2=1906.925, df=149, χ^2/df=12.798, CFI=.879, IFI=.879, RMSEA=.094, SRMR=.058. Model 2 correlated errors: χ^2=649.486, df=134, χ^2/df=4.847, CFI=.965, IFI=.965, RMSEA=.053, SRMR=.044. Model 3 second-order: χ^2=618.766, df=131, χ^2/df=4.652, CFI=.967, IFI=.967, RMSEA=.052, SRMR=.043. Note: "Article reports df = 131 in Table 1 but df = 133 in Figure 2."	Standardized loadings in figures approximately .53–.91; mean not reported.	Observed correlations: PB–WB=.814; PB–patient=.474; WB–patient=.616. Discriminant validity weak for PB–WB: shared variance r^2=.94 in CFA-based assessment.	Model 2 added multiple correlated errors. Explicitly reported pairs included PB items CBI1–CBI2, CBI3–CBI4, CBI1–CBI5, CBI2–CBI5, and additional pairs CBI10–CBI11, CBI15–CBI16, CBI1–CBI7, CBI2–CBI14, CBI5–CBI7, CBI8–CBI10, CBI9–CBI10, CBI12–CBI15 and CBI14–CBI16. No items removed.	Cronbach α: PB=.88; WB=.86; patient=.85; global burnout=.905. AVE: PB=.52; WB=.48; patient=.49.	DASS-21 stress, depression and anxiety; Sheehan Disability Scale (SDS) work/social/affective life impairment; Ruminative Response Scale; Satisfaction With Life Scale.	Provides support for a global burnout index in physicians, while retaining the three domains for more detailed interpretation.
21	Aiello, Fiabane, Margheritti, Magnone, Bolognini, Miglioretti & Giorgi (2022) — Psychometric properties of the Copenhagen Burnout Inventory (CBI) in Italian Physicians. La Medicina del Lavoro, 113(4), e2022037. DOI: 10.23749/mdl.v113i4.13219	Italian CBI for physicians; adapted 18-item version with personal, work-related and client/patient-related burnout; item 17 removed before analysis because of deontological/social-desirability concerns.	Cross-sectional web-based psychometric standardization study; Italian physicians delivering medical care, N=915.	Original three-factor structure retained for the 18-item adapted CBI.	CFA: CFI=.94; TLI=.93; RMSEA=.09; SRMR=.04.	Text loading ranges: PB=.67–1.02; WB=.59–1.03; CB=.70–1.02. Standardized figure loadings approximately .59–.92; mean≈.80.	Figure indicates high factor correlations: PB–WB≈.94; PB–CB≈.64; WB–CB≈.78.	No freed residuals reported. Removed/adapted out item 17 ('frustrating to work with clients/patients') before analysis.	Cronbach α: total=.96; PB=.92; WB=.91; CB=.90. Item-total correlations: total=.51–.84; PB=.74–.88; WB=.59–.83; CB=.56–.83.	PHQ-8 depression; GAD-7 anxiety; General Self-Efficacy Scale (GSE); diagnostic classification based on co-occurring PHQ-8/GAD-7≥10 and low GSE.	Strong Italian physician evidence; high PB–WB association again suggests overlap between personal and work-related exhaustion.
22	Jeon, You, Kim, Kim & Cho (2019) — Psychometric properties of the Korean version of the Copenhagen Burnout Inventory in Korean homecare workers for older adults. PLOS ONE, 14(8), e0221323. DOI: 10.1371/journal.pone.0221323	Korean CBI (CBI-K) for homecare workers; original three scales refined; also proposed a shorter two-factor PB+CRB version.	Cross-sectional construct-validation study; Korean homecare workers for older adults, N=464/465. Study population N = 465; CFA/model-fit analyses N = 464.	Original three-factor model inadequate. Refined 18-item three-factor model accepted; refined 12-item two-factor PB+CRB model recommended fo practical use because WRB correlated too highly with PB and CRB.	Original 19-item three-factor: CFI=.866, SRMR=.056, RMSEA=.112. Refined 18-item three-factor: CFI=.924, SRMR=.049, RMSEA=.091. Refined 12-item two-factor: CFI=.950, SRMR=.047, RMSEA=.084.	All 18 retained items in the refined model had λ≥.50; mean not reported. Removed WRB item 4 had very low loading λ=.16/.19.	PB–CRB=.74 in refined two-factor model. WRB correlations with PB and CRB were >.85, indicating poor discriminant validity.	Removed WRB item 4 (reverse 'energy for others' item). Freed residuals: PB items 1–2; WRB items 6–7; CRB items 1–2.	Composite reliability: PB=.91; WRB=.92; CRB=.90. Cronbach α not reported in extracted text.	CESD-10 depressive symptoms; work–life conflict and sociodemographic/work covariates.	Supports Korean CBI use with homecare workers but suggests WRB may be redundant in this occupational setting.

23	Kováč & Lisá (2025). Comparing burnout among client facing and non client facing employees using the Copenhagen burnout inventory. Discover Public Health, 22, 604.DOI: 10.1186/s12982-025-01003-9	CBI, 19 items; PB, WB, CB	Large cross-sectional occupational sample, N=34,727; CFA, invariance, network analysis, group comparisons.	Three-factor CBI confirmed; measurement invariance across gender and age supported at acceptable fit levels.	CFA: χ^2(149)=20,956.16, p<.001; CFI=.916; TLI=.904; RMSEA=.077 [90% CI .076,.078]; SRMR=.044; GFI=.905. Gender invariance: CFI=.902–.909; TLI=.895–.904; RMSEA=.076–.080; SRMR=.044–.049. Age invariance: CFI=.890–.908; TLI=.894–.903; RMSEA=.077–.081; SRMR=.050–.055.	Standardized loadings .536–.819; mean NR.	Latent covariances: F1–F2=.794; F1–F3=.582; F2–F3=.712.	Freed residuals / removed items: NR; no item deletion reported.	F1 ω=.870 (α=.866); F2 ω=.882 (α=.868); F3 ω=.886 (α=.879); overall ω=.935, α=.928.	Client-facing status, gender, age, occupational role; network centrality indices.	Strong sample size supports stable estimates, but fit is acceptable rather than excellent.
24	Wongtrakul et al. (2021). Reliability and validity study of the Thai adaptation of the Copenhagen Burnout Inventory-Student Survey (CBI-SS) among preclinical medical students at the Faculty of Medicine Siriraj Hospital, Mahidol University, Thailand. PLOS ONE, 16(12), e0261887.DOI: 10.1371/journal.pone.0261887	0	Translation/adaptation and validation; N=414; EFA/CFA, test–retest.	Four-factor Thai CBI-SS accepted after removal of items 6, 10 and 17.	CFA: χ^2/df=2.39; CFI=.957; GFI=.909; RMSEA=.058; TLI=.949; NFI=.928.	EFA retained loadings ≈ .568–.905; mean ≈ .79. CFA standardized loadings ≈ .52–.90.	PB–SRB=.82; PB–CRB=.40; PB–TRB=.33; SRB–CRB=.43; SRB–TRB=.44;	Freed residuals:Removed items: 6 (weak/susceptible to illness), 10 (energy for family/friends), 17 (give more than get back from colleagues) due loadings <.50. Cor-Risiduals: PBI1/PB2=0.38; PB1/PB3=.25; SB14/SB11 =-.31; SB18/SB19=.30; TB23/TB24=.27	Total α=.929; PB=.898; study-related=.896; colleague-related=.910; teacher-related=.900. Test–retest: .820, .870, .821, .787. AVE/CR: PB .626/.893; SRB .601/.899; CRB .677/.913; TRB .606/.900.	Primarily psychometric; no external criterion variables beyond stability and discriminant validity.	Consistent with recurring weakness of the reverse/energy item. PB-SRB overlap high
25	Cordero-Franco et al. (2022). Validation of the Spanish Version of the Copenhagen Burnout Inventory in Mexican Medical Residents. Archives of Medical Research, 53(6), 617–624.DOI: 10.1016/j.arcmed.2022.08.001	Spanish CBI, 19 items; PB, WB, patient-related burnout	Translation/validation, online cross-sectional survey, N=525 residents.	EFA suggested two factors; CFA showed the original three-factor model had adequate fit and was retained despite imperfect discrimination between PB and WB.	Original 3-factor: RMSEA=.08; CFI=.95; TLI=.94; SRMR=.04. 3-factor minus one item: RMSEA=.08; CFI=.95; TLI=.94; SRMR=.04. 2-factor minus one item: RMSEA=.10; CFI=.93; TLI=.91; SRMR=.04.	EFA loadings .19–.89; all except work item 4/item 13 >.50.	PB and WB did not show good discrimination; PB-WB = .91; PB-CB = .57; WB-CB=0.66	Freed residuals: PB1-PB2 = .49. No final removal; low item noted: work-related reverse energy item (item 13 / work item 4) loading ≈ .19; deletion did not materially improve model.	α: PB=.94; WB=.95; patient=.93.	Oldenburg Burnout Inventory (OLBI); General Work Stress Scale; content validity by expert panel.	Supports reliability and criterion validity, but construct validity between PB and WB was not fully consistent.
26	Nguyen et al. (2025). Psychometric properties of the Vietnamese version of the Copenhagen Burnout Inventory (CBI-V) and its associated factors among community pharmacists in Thua Thien Hue, Vietnam. Journal of Health Psychology, 30(7), 1577–1592.DOI: 10.1177/13591053251314932	Vietnamese CBI-V, 19 items; PB, WB, CB	Psychometric assessment and associated factors; N=362 community pharmacists.	Original 19-item three-factor CBI retained; best model allowed selected correlated errors. Alternative 18-item model without CB4 did not improve fit sufficiently.	Model 1 original: RMSEA=.091; GFI=.841; AGFI=.798; TLI=.890; CFI=.904; NFI=.877; CMIN/df=4.001. Model 2 with correlated errors: RMSEA=.069; GFI=.897; AGFI=.864; TLI=.937; CFI=.947; NFI=.919; CMIN/df=2.723. Model 3 without CB4: RMSEA=.071; GFI=.899; AGFI=.866; TLI=.939; CFI=.949; NFI=.923; CMIN/df=2.812.	Standardized CFA loadings NR in parsed text; corrected item-total/discrimination values≈ .502–.818.	PB–WB=.80; PB–CB=.62; WB–CB=.77.	Freed residuals in final model: WB1–WB2, WB4–WB6, PB1–PB4, PB2–PB3, CB4–CB5. Removed items: none in accepted model; CB4 tested for removal.	α/ω: PB=.904/.904; WB=.897/.896; CB=.867/.867.	Known-groups and regression with working site, education, position, experience, job satisfaction, CME, accurate drug-information sources, phone/Zalo consultations.	Fit improved mainly through local residual covariances rather than deletion.
27	Fong et al. (2014). Psychometric Properties of the Copenhagen Burnout Inventory—Chinese Version. The Journal of Psychology, 148(3), 255–266.DOI: 10.1080/00223980.2013.781498	Chinese CBI-C, 19 items; PB, WB, CB	Psychometric validation with follow-up; baseline N=312, follow-up N=245.	Revised three-factor model accepted after freeing three correlated residuals; cross-validated at 4 months.	Original 3-factor baseline: χ^2=471.27 df=149; CFI=.969; TLI=.964; RMSEA=.083 [.075,.092]; WRMR=1.177. Revised 3-factor: χ^2=316.28 df=146; CFI=.984; TLI=.981; RMSEA=.061 [.052,.070]; WRMR=.910. Revised follow-up: χ^2=354.92 df=146; CFI=.986; TLI=.983; RMSEA=.076 [.066,.087]; WRMR=.891.	All loadings except item 13 > .50; exact range/mean NR in parsed text.	Latent factor correlations ≈ .73–.95. Test–retest subscale correlations ≈ .60–.63.	Freed residuals: items 14–15, 11–12, 1–2. Removed items: none; item 13 low but retained.	α: PB=.91; WB=.84; CB=.78.	Age, physical distress, anxiety, depression, workplace social support, work commitment.	Strong criterion evidence; local residual dependencies were important for fit.
28	Milfont et al. (2008). Burnout and Wellbeing: Testing the Copenhagen Burnout Inventory in New Zealand Teachers. Social Indicators Research, 89, 169–177.DOI: 10.1007/s11205-007-9229-9	CBI, 19 items; PB, WB, student-related burnout	Validation and well-being association study; N= 205 eligible full-time teachers; final analytic sample N = 129; response rate = 62.9%.	One-factor subscale models, correlated three-factor model, higher-order model and two-factor alternative all considered acceptable; very high PB–WB overlap noted.	Subscale models: PB χ^2=22.69 df=9, CFI=.97, RMSEA=.11, SRMR=.052; WB χ^2=19.97 df=14, CFI=.99, RMSEA=.058, SRMR=.042; student χ^2=10.58 df=9, CFI=.99, RMSEA=.037, SRMR=.038. Three-factor/higher-order: χ^2≈360.55 df=149, CFI=.96, RMSEA =.11, SRMR=.067.	PB .58–.78 (mean=.71); WB .53–.85 (mean=.69); student .43–.75 (mean=.63).	Observed: PB–WB=.87; PB–student=.75; WB–student=.75. Latent: PB–WB≈1.01; PB–student=.90; WB–student=.94.	Freed residuals / removed items: none.	PB α=.87; WB α=.87; student α=.79; general α=.94.	WHO-5 Well-Being Index, school connection, perceived general health; controlled age/sex.	Demonstrates criterion validity but also near-redundancy of PB and WB in this sample.
29	Montgomery et al. (2021). Psychometric properties of Copenhagen Burnout Inventory among nurses. Research in Nursing & Health, 44(2), 308–318.DOI: 10.1002/nur.22114	CBI, 19 items; PB, WB, CB	Psychometric validation; statewide hospital nurse survey, N=928.	Three-factor ordinal CFA accepted with two correlated residuals; item 10 retained despite low explained variance.	CFA: χ^2(147)=1140.47, p<.001; χ^2/df=7.76; RMSEA=.087 [90% CI .083,.092]; CFI=.996; TLI=.995; SRMR=.054. Removing item 10 worsened fit: χ^2/df=8.28; RMSEA=.091; CFI=.996; TLI=.995; SRMR=.055.	Standardized loading equivalent from R^2 ≈ .45–.94; R^2 range .206–.878. Corrected item correlations .40–.86.	PB–WB=.856; PB–CB=.623; WB–CB=.731.	Freed residuals: items 16–17 and 14–15. Removed: none; item 10 low R^2=.206 but retained.	α: PB=.91; WB=.89; CB=.92.	PES-NWI nurse work environment, job satisfaction, intent to leave.	Strong reliability and convergent validity; local dependence among client-burnout items.
30	Oluwadiya et al. (2024). Evaluating the factor structure, reliability and validity of the Copenhagen Burnout Inventory-Student Survey (CBI-SS) among faculty of arts students of Ekiti State University, Ado-Ekiti, Nigeria. Scientific Reports, 14, 10476.DOI: 10.1038/s41598-024-61310-0	CBI-SS; final 24 items; PB, SRB, faculty/study-related, lecturer-related burnout	CFA validation; N=635.	Four-factor CBI-SS accepted after removing item 10 due very low loading/AVE problem.	χ^2/df=1.42; CFI=.996; TLI=.995; RMSEA=.026 [95% CI .019,.035]; SRMR=.050.	After item 10 removal: PB .697–.896; SRB .540–.873; FSRB .712–.884; LRB .726–.837. Item 10 loading=.123 before removal.	PB–SRB=.663; PB–FSRB=.527; PB–LRB=.507; SRB–FSRB=.637; SRB–LRB=.686; FSRB–LRB=.713.	Freed residuals: none reported. Removed item 10.	Total ω=.957; subscales ω=.862–.914. Total α=.949; subscales α=.864–.915.	General Academic Self-Efficacy Scale (GASE), CGPA, self-reported burnout, perceived course stress.	Excellent fit; again reverse/energy item 10 was problematic.
31	Bolatova et al. (2021). Psychometric Properties of the Copenhagen Burnout Inventory in a Sample of Medical Students in Kazakhstan. Psychology in Russia: State of the Art, 14(2), 15–24.DOI: 10.11621/pir.2021.0202	Russian CBI-Student Survey; PB, SRB, CRB, TRB	Validation; N=771 medical students; CFA, reliability, validity.	Four-factor R-CBI-S accepted; one-factor model rejected.	Four-factor: χ^2/df=3.881; RMSEA=.0611; CFI=.940; TLI=.933. One-factor: χ^2/df=17.963; RMSEA=.148; CFI=.638; TLI=.605. KMO=.947; Bartlett p<.001.	Factor loadings approx .483–.918; mean ≈ .75.	PB–SRB=.0.880; PB–CRB=.0.318; PB–TRB=.0.577; SRB–CRB=.380; SRB–TRB=.707; CRB–TRB=.441.	Freed residuals / removed items: none reported.	Total α=.94; PB=.896; SRB=.884; CRB=.874; TRB=.926; ICC=.81. AVE/CR: PB .52/.87; SRB .50/.87; CRB .51/.86; TRB .56/.88.	Satisfaction with study, GAD-7 anxiety, PHQ-9 depression.	Good support for four-factor student model.
32	Fiorilli et al. (2015). Copenhagen Burnout Inventory (CBI): A validation study in an Italian teacher group. TPM - Testing, Psychometrics, Methodology in Applied Psychology, 22(4), 537–551.DOI: 10.4473/TPM22.4.7	Italian CBI; final 17 items; PB, WB, student-related burnout	Validation; N=1,497 teachers; CFA and criterion correlations.	Three correlated factors accepted after removing WB1 and WB6.	M1 1-factor: χ^2=3291.2 df=153; RMSEA=.12; SRMR=.07; CFI=.79. M2 uncorrelated: χ^2=3710.1 df=143; RMSEA=.13; SRMR=.32; CFI=.72. M3 correlated: χ^2=1205.4 df=140; RMSEA=.07; SRMR=.04; CFI=.93. Final M3a: χ^2=591.8 df=105; RMSEA=.06; SRMR=.04; NFI=.95; TLI=.95; CFI=.96.	Final loadings .42–.88; mean ≈ .68.	Latent correlations approx .76, .86 and .92. Observed scale correlations: WB–PB=.769; WB–CB=.732; PB–CB=.624.	Freed residuals: none. Removed WB1 and WB6 due excessive standardized residuals.	PB α=.89, composite=.89; WB α=.78, composite=.78; student α=.78, composite=.79.	UWES (vigor, absorption, dedication); self-efficacy.	Clear example of item removal improving fit in teacher context.
33	Hernández Breña et al. (2022). Translation, adaptation, and validation of the Copenhagen Burnout Inventory for the Peruvian version. Revista de Psicología, 40(2), 711–738.DOI: 10.18800/psico.202202.003	Peruvian Spanish CBI; PB, WB, CB	Translation/adaptation validation; two waves, n=160; CFA, reliability, convergent/test-criterion validity.	Three correlated factors accepted; one- and two-factor alternatives tested.	1-factor: χ^2(152)=469.279, p<.001; RMSEA=.12; SRMR=.09; CFI=.95; TLI=.95. 2-factor: χ^2(151)=411.471; RMSEA=.10; SRMR=.08; CFI=.96; TLI=.96. 3-factor: χ^2(149)=259.355; RMSEA=.07; SRMR=.06; CFI=.98; TLI=.98.	Standardized loadings .41–.92; mean NR.	Scale correlations .55–.81; PB–WB=.81; PB–CB=.55; WB–CB=.66.	Freed residuals / removed items: NR.	Trial 1 α: PB=.87; WB=.88; CB=.82. Trial 2 α=.87/.89/.81. Omega=.92/.92/.91. ICC=.66/.73/.69.	Secondary Traumatic Stress Scale, Perceived Stress Scale, Fear of COVID-19, self-reported health, workload.	Good evidence for three-factor model; PB–WB again high.
34	Johnson (2024). Use of the Copenhagen Burnout Inventory among US academic librarians: Examining construct validity through factor structure and model fit. The Journal of Academic Librarianship, 50(5), 102922.DOI: 10.1016/j.acalib.2024.102922	CBI, 19 items; PB, WB, client-related burnout	CFA construct validation in probability sample; complete CFA N=372.	Correlated three-factor model accepted as adequate; two-factor work/client and three single-factor subscale models also fit reasonably.	Three-factor: χ^2(149)=574.991, p<.001; CFI=.918; SRMR=.058; RMSEA=.088. Two-factor work/client: χ^2(64)=177.479; CFI=.962; SRMR=.052; RMSEA=.069. Single PB: χ^2(9)=36.483; CFI=.979; SRMR=.028; RMSEA=.091. Single WB: χ^2(14)=65.255; CFI=.969; SRMR=.033; RMSEA=.099. Single CB: χ^2(9)=25.325; CFI=.987; SRMR=.023; RMSEA=.070.	All loadings >.40; lowest=.532 (client item 4). Corrected item-total correlations .532–.867.	Scale correlations PB–WB=.97; PB–CB=.48; WB–CB=.53.	Freed residuals / removed items: none. Client item 4 flagged but retained.	α: PB=.896; WB=.912; CB=.889.	No external criterion variables; construct validity through CFA/model comparison and CTT item diagnostics.	Supports use in librarianship but criterion validity still needed. PB-WB overlap very high
35	Thrush et al. (2021). Psychometric properties of the Copenhagen Burnout Inventory in an academic healthcare institution sample in th U.S. Evaluation & the Health Professions, 44(4), 400–405.DOI: 10.1177/0163278720934165	CBI, 19 items; PB, WB, patient-related burnout	Secondary psychometric analysis; N=1,679 healthcare employees; CFA, invariance, discriminant validity.	Original three-factor model supported; configural and metric invariance across professional role, age and gender; scalar/residual not established.	χ^2(149)=1891.13, p<.0001; CFI=.922; RMSEA=.083; SRMR=.044. Invariance models: ΔCFI < .01 for configural/metric across status, age, gender.	Standardized loadings .57–.86; mean NR.	WB–PB=.96; patient–WB=.67; patient–PB=.58.	Freed residuals / removed items: none reported.	Overall α=.946; PB α=.892; WB α=.896; patient α=.897.	Meaningful work scale / WAMI subset; professional status, age, gender for invariance.	Strong US healthcare support; high PB–WB correlation and only partial invariance beyond metric.
36	Yeh et al. (2007). Psychometric Properties of the Chinese Version of Copenhagen Burnout Inventory Among Employees in Two Companies in Taiwan. International Journal of Behavioral Medicine, 14(3), 126–133.DOI: 10.1007/BF03000183	Chinese C-CBI; selected PB and WB scales only (13 items)	Psychometric validation among employees from two companies, N=384; EFA and criterion correlations.	EFA extracted two empirical factors, but PB and WB scales were highly correlated and overlapping.	EFA/PCA Promax: two factors; variance explained F1=59%, F2=8%. CFA fit indices not reported.	EFA loadings reported ≥.30: PB items .67–.99; WB items .40–.84. Item 13 energy-for-others loading .42. Mean NR.	PB–WB r=.78.	Freed residuals: NR. No items removed, but item 13 correlated poorly with other items and behaved differently by gender.	Cronbach α > .86 for both PB and WB in men and women subgroups.	Working hours, C-JCQ psychological demands/job control/support, ERI over-commitment, SF-36 mental health/vitality/general health, job satisfaction.	Early evidence of PB–WB overlap and problematic reverse/energy item.

PB/PRB	Personal burnout / personal-related burnout
WB/WRB/SRB	Work-related burnout; in student versions, study/studies-related burnout
CB/CRB/TRB	Client-related, colleague-related or teacher-related burnout depending on version
CBI-S / CBI-SS	Copenhagen Burnout Inventory—Student Version / Student Survey
CFA/EFA	Confirmatory / Exploratory factor analysis
SOHM	Second-order hierarchical model
CFI/TLI/RMSEA/SRMR	Common global model fit indices
CR/AVE/α/ω	Composite reliability, average variance extracted, Cronbach alpha, McDonald omega
NR	Not reported in the article text or not applicable
PB	Personal burnout
WB	Work-related burnout
CB	Client-related / patient-related burnout
CBI	Copenhagen Burnout Inventory
CBI-SS / CBI-S	Copenhagen Burnout Inventory – Student Survey / Student version
SRB	Study-related burnout
CRB	Colleague-related burnout or client-related burnout, depending on article context
TRB	Teacher-related burnout
CFA	Confirmatory factor analysis
EFA	Exploratory factor analysis
PCA	Principal components analysis
RMSEA	Root mean square error of approximation
SRMR	Standardised root mean square residual
CFI	Comparative fit index
TLI	Tucker–Lewis index
GFI / AGFI / NFI	Goodness-of-fit index / adjusted GFI / normed fit index
α	Cronbach's alpha reliability coefficient
ω	McDonald's omega reliability coefficient
CR	Composite reliability
AVE	Average variance extracted
NR	Not reported in the article or not extracted

Table S2: Trend summary from the evidence matrix of published EFA/CFA models in CBI validation studies

Theme	Overall trend summary	Implication for CBI interpretation
Dominant factor structure	Most formal validation studies supported the intended three-factor CBI structure, or the four-factor student adaptation (CBI-S/CBI-SS). However, some healthcare and student studies found PB and WB to be very highly correlated or even collapsed into one broader exhaustion factor.	Use PB, WB and CB as conceptually distinct domains, but interpret PB–WB separation cautiously, especially in healthcare and education samples.
Model fit	CFA fit was usually acceptable after item refinement or freeing a small number of residual covariances. The strongest fits often followed correlated residuals between adjacent/similarly worded items or removal of reversed items.	Good global fit does not necessarily imply item-level simplicity; inspect local dependence and problematic items.
Repeated item problems	Reverse/energy item 10 or item 13 depending on numbering/version ("energy for family and friends during leisure time") repeatedly showed weak, negative, or anomalous loading/item-total behavior in Brazil, Colombia, Latvia, Serbia and student versions.	Consider reviewing reverse scoring, translation, and cultural interpretation before retaining this item; report sensitivity analyses with and without it.
Reliability	Internal consistencies were consistently adequate to excellent, mostly α/ω > .70 and often > .90. Short forms retained acceptable reliability.	CBI domains are reliable; very high reliability may also reflect item redundancy, particularly within PB/WB.
External validity	Burnout correlated positively with distress, emotional demands, role conflict, anxiety/depression/stress, and negatively with vitality, mental health, general health, work engagement, well-being and job satisfaction.	Criterion validity is strongest for exhaustion/distress and well-being outcomes; client-related burnout often shows weaker or more context-specific external associations.
Sector/population sensitivity	Sector-specific adaptations sometimes changed the number of retained items and residual correlations. Student versions require four-domain scoring and cross-cultural invariance may not hold.	Do not assume universal invariance. Test measurement invariance before comparing cultures, sectors or professional roles.
CBI-SS student structure	Student versions generally supported four dimensions (personal, study-related, colleague/faculty/teacher/lecturer-related). Studies in Thailand, Nigeria, Kazakhstan and Morocco found adequate reliability and fit, but item deletions were common.	CBI-SS appears useful across student contexts, but local adaptation and item screening are essential.
Local residual dependence	Several studies improved fit by freeing residual correlations between semantically similar items (e.g., Fong, Nguyen, Montgomery). (e.g. PB1-PB2)	Report item pairs with freed residuals transparently; do not treat improved global fit as proof of clean simple structure.
Cross-cultural adaptation	Translations/adaptations in Thai, Spanish, Vietnamese, Chinese, Russian, Peruvian Spanish and others generally supported the CBI/CBI-SS, but item-level behaviour varied by language, sector and role.	Do not assume cross-cultural equivalence from acceptable reliability alone; inspect loadings, item residuals, DIF, and invariance.
Overall conclusion	The evidence supports the CBI as a flexible, reliable and useful burnout measure, but the literature repeatedly shows PB–WB overlap, local residual dependencies (PB1-PB2), and reverse-item problems.	For research: report CFA/EFA details, residual covariances, removed items and sensitivity analyses. For practice: subscale scores are usually more defensible than a single total score unless a hierarchical bifactor model is explicitly supported.

File S1: Copenhagen Burnout Instrument

Please rate the following questions as per the response categories indicated:

1= "always"	2= "often"	3= "sometimes"	4= "seldom"	5= "never/almost never"
Scoring 100	Scoring 75	Scoring 50	Scoring 25	Scoring 0

1. How often do you feel tired?
2. How often are you physically exhausted?
3. How often are you emotionally exhausted?
4. How often do you think: "I can't take it anymore"?
5. How often do you feel worn out?
6. How often do you feel weak and susceptible to illness?

1= "to a very high degree"	2= "to a high degree"	3= "somewhat"	4= "seldom"	5= "to a very low degree"
Scoring 100	Scoring 75	Scoring 50	Scoring 25	Scoring 0

7. Is your academic work emotionally exhausting?
8. Do you feel burnt out because of your academic work?
9. Does your academic work frustrate you?

1= "always"	2= "often"	3= "sometimes"	4= "seldom"	5= "never/almost never"
Scoring 100	Scoring 75	Scoring 50	Scoring 25	Scoring 0

10. Do you feel worn out at the end of the day after academic work?
11. Are you exhausted in the morning at the thought of another day working on your studies?
12. Do you feel that every academic working hour is tiring for you?
13. Do you have enough energy for family and friends during leisure time?

1= "to a very high degree"	2= "to a high degree"	3= "somewhat"	4= "seldom"	5= "to a very low degree"
Scoring 100	Scoring 75	Scoring 50	Scoring 25	Scoring 0

14. Do you find it hard to work with peers?
15. Do you find it frustrating to work with peers?
16. Does it drain your energy to work with peers?
17. Do you feel that you give more than you get back when you work with peers?

1= "always"	2= "often"	3= "sometimes"	4= "seldom"	5= "never/almost never"
Scoring 100	Scoring 75	Scoring 50	Scoring 25	Scoring 0

18. Are you tired of working with peers?
19. Do you sometimes wonder how long you will be able to continue working with peers?

Adapted from Kristensen et al., (2005).

Kristensen, T. S., Borritz, M., Villadsen, E., & Christensen, K. B. (2005). The Copenhagen Burnout Inventory: A new tool for the assessment of burnout. *Work & Stress*, *19*(3), 192–207. https://doi.org/10.1080/02678370500297720

File S2: Study Demands and Study Resources Scale

Study demands scale

Please rate the statements below on a scale from 1="never" to 4="always"

1. Do you have too much work to do within the module?
2. Do you work under time pressure?
3. Do you have to work extra hard to complete something?
4. Do you have to give continuous attention to your studies within the module?
5. Do you have to remember many things in your studies for the module?

Study resources scale

Please rate the statements below on a scale from 1="never" to 4="always"

1. Do your studies in the module require creativity?
2. Do your studies within the module make sufficient demands on your skills and capacities?
3. Do you have enough variety in your studies in the module?
4. Do your studies in the module offer you opportunities for personal growth and development?
5. Do your studies give you the feeling that you can achieve something?
6. Do your studies in the module give you the opportunity for independent thought and action?
7. Can you count on your fellow students when you run into difficulties in your studies for the module?
8. If necessary, can you ask your fellow students for help?
9. Do you get on well with your fellow students?
10. Can you count on your lecturer if you run into difficulties in your studies?
11. Do you get on well with your lecturer?
12. Do you know exactly what your lecturer expects of you in your studies?
13. Do you know exactly what your lecturer thinks of your performance?
14. Do you receive sufficient information on the results of your studies for the module?
15. Can you discuss study problems with your lecturer?
16. Are you kept adequately up-to-date about issues within your module/faculty?
17. Is the decision-making process of your faculty/university clear to you?
18. Is it clear to whom you should address within the faculty/university if you experience specific problems?

Adapted from Mokgele (2014).

Mokgele, K. R. F. (2014). *Study demands, study resources and well-being of first-year students in South African higher education institutions* [Ph.D. thesis, North-West University]. Available online: http://hdl.handle.net/10394/14717

Table S3: Value-added ratio of a factor score (VAR)

Dueber and Toland (2023, pp. 228-229)

Cut-off points for predicting VAR ≥ 1.10

Explanation:

The following table provides accurate cut-off points from Dueber and Toland (2023) for predicting VAR ≥ 1.10 for ECVss and ωHS for three subfactors, as shown in Table 3 under section 4.3. Cut-off points for four subfactors in Table 5 are reported in Dueber and Toland (2023, pp. 228-229)

ECVss	ωS
0.05	0.987
0.10	0.953
0.15	0.913
0.20	0.868
0.25	0.818
0.30	0.761
0.35	0.699
0.40	0.631
0.45	0.558
0.50	0.479

ωS	ωHS
0.50	0.257
0.55	0.251
0.60	0.245
0.65	0.236
0.70	0.226
0.75	0.211
0.80	0.192
0.85	0.161
0.90	0.102
0.95	0.000

Dueber, D. M., & Toland, M. D. (2023). A bifactor approach to subscore assessment. *Psychological Methods*, *28*(1), 222–241. https://doi.org/10.1037/met0000459.

Table S4: Study Demands and Resources Scale factor models

	Bifactor PSEM$^{C:}$ for Study Resources						Study-demands		
**ITEM**	**Gf**	**F1**	**F2**	**F3**	**F4**	**Res**	**ITEM**	1-Fact	Res
R1	**0.10**	**0.52**	-0.04	0.03	0.00	0.72	V1	0.747	0.44
R2	**0.24**	**0.64**	0.00	0.01	0.03	0.54	V2	0.715	0.48
R3	**0.49**	**0.31**	-0.01	0.10	-0.06	0.66	V3	0.703	0.50
R4	**0.66**	**0.36**	-0.03	0.01	-0.31	0.34	V4	0.457	0.79
R5	**0.76**	**0.16**	-0.03	-0.03	-0.51	0.14	V5	0.377	0.85
R6	**0.65**	**0.27**	-0.03	0.01	-0.32	0.40			
R7	**0.30**	0.00	**0.79**	0.02	0.03	0.29			
R8	**0.23**	-0.02	**0.86**	0.00	0.02	0.21			
R9	**0.37**	-0.05	**0.56**	0.03	0.00	0.55			
R10	**0.62**	0.00	0.07	**0.36**	0.02	0.48			
R11	**0.67**	0.06	0.03	**0.45**	-0.03	0.35			
R12	**0.62**	0.03	0.02	**0.45**	0.00	0.41			
R13	**0.80**	-0.09	0.03	**0.13**	0.23	0.28			
R14	**0.84**	-0.12	0.00	0.12	**0.11**	0.23			
R15	**0.72**	0.00	-0.01	**-0.01**	0.23	0.45			
R16	**0.60**	0.08	-0.03	-0.03	**0.26**	0.57			
R17	**0.66**	0.03	0.00	0.15	**0.14**	0.52			
R18	**0.73**	-0.01	0.03	-0.01	**0.28**	0.39			
SEAVE	0.02	0.03	0.02	0.03	0.03	0.03		0.02	0.03
SEMAX	0.03	0.05	0.03	0.05	0.05	0.05		0.03	0.03
Correl^R:									
V4/V5								0.23	

Notes: 1-Fact = One-factor CFA model; Gf= General resources factor; F1= Growth, F2= Peer Support; F3=Lecturer Support; F4= Information Accessibility; SEAVE = Mean standard errors for item loading estimations; SEmax = Maximum standard error for item loading estimations; Correl^R = includes correlated residuals, Bolded values = Primary factors and statistically significant ($p \leq 0.05$), Underlined values = substantive cross-loadings and statistically significant ($p \leq 0.05$); ^{C} = includes cross-loadings with ALF (0,1) priors.
